# The Potential of Amphiphilic Cyclodextrins as Carriers for Therapeutic Purposes: A Short Overview

**DOI:** 10.3390/pharmaceutics17081086

**Published:** 2025-08-21

**Authors:** Ramona Daniela Pârvănescu, Marius Păpurică, Ionica Oana Alexa, Cristina Adriana Dehelean, Codruța Șoica, Elena Alina Moacă, Adriana Ledeți, Mirela Voicu, Dorina Coricovac, Cristina Trandafirescu

**Affiliations:** 1 Research Center for Experimental Pharmacology and Drug Design, Faculty of Pharmacy, “Victor Babeș” University of Medicine and Pharmacy Timișoara, Eftimie Murgu Square No. 2, RO-300041 Timișoara, Romania; ramona.parvanescu@umft.ro (R.D.P.); oana.mutu@umft.ro (I.O.A.); codrutasoica@umft.ro (C.Ș.); trandafirescu.cristina@umft.ro (C.T.); 2 University Department of Pharmaceutical Chemistry, Faculty of Pharmacy, “Victor Babeș” University of Medicine and Pharmacy Timișoara, Eftimie Murgu Square No. 2, RO-300041 Timișoara, Romania; 3 University Clinic of Anaesthesia and Intensive Care, Department X Surgery II, “Victor Babeș” University of Medicine and Pharmacy Timișoara, RO-300041 Timișoara, Romania; papurica.marius@umft.ro; 4 Research Center for Pharmaco-Toxicological Evaluations, Faculty of Pharmacy, “Victor Babeș” University of Medicine and Pharmacy Timișoara, Eftimie Murgu Square No. 2, RO-300041 Timișoara, Romania; cadehelean@umft.ro (C.A.D.); alina.moaca@umft.ro (E.A.M.); dorinacoricovac@umft.ro (D.C.); 5 University Clinic of Toxicology, Drug Industry, Management and Legislation, Faculty of Pharmacy, “Victor Babeș” University of Medicine and Pharmacy Timișoara, Eftimie Murgu Square No.2, RO-300041 Timișoara, Romania; 6 University Department of Pharmacology-Pharmacotherapy, Faculty of Pharmacy, “Victor Babeș” University of Medicine and Pharmacy Timișoara, Eftimie Murgu Square No. 2, RO-300041 Timișoara, Romania; 7 Advanced Instrumental Screening Center, Faculty of Pharmacy, “Victor Babeș” University of Medicine and Pharmacy Timișoara, Eftimie Murgu Square No. 2, RO-300041 Timișoara, Romania; 8 University Department of Analytical Chemistry, Faculty of Pharmacy, “Victor Babeș” University of Medicine and Pharmacy Timișoara, Eftimie Murgu Square No. 2, RO-300041 Timișoara, Romania

**Keywords:** amphiphilic cyclodextrins, inclusion complexes, solubilising agents, cancer treatment, gene delivery

## Abstract

Cyclodextrins, since their discovery in the late 19th century, have gained tremendous interest in biomedical research, beginning with their recognition as safe pharmaceutical excipients, and continuing with exploiting their potential for enhancing the therapeutic response of active pharmaceutical ingredients, and also to be used as drugs for specific medical purposes. This review presents an integrative perspective on amphiphilic cyclodextrins, the manuscript being divided into two parts, one devoted to the properties of amphiphilic cyclodextrins, while the second one is dedicated to their biomedical applications, with an emphasis on cancer therapy.

## 1. Introduction

Cyclodextrins (CDs) were discovered in 1891 by Antoine Villiers as crystalline dextrins, produced by the enzymatic degradation of potato starch by *Bacillus amylobacter*. Schardinger described, at the beginning of the 20th century, the preparation, separation, and purification of dextrin A and B. He indicated that they were cyclic polysaccharides, as a conclusion of his research. The structure of CDs was described by Freudenberg 30 years later, and, at the end of the 1940s, Cramer, based on his studies regarding natural cyclodextrins, used the word “cyclodextrin” to define the dextrin’s characteristics. Cramer also studied the inclusion complex formation between CDs and guest molecules. The first marketed pharmaceutical product was prostaglandin E2/β-CD in 1976 in Japan [1,2].

Cyclodextrins are toroidal molecules with complexing capacity and can be used as carriers of different substances; they are biocompatible, biodegradable, and have a relatively low cost of production. Due to these favourable characteristics, CDs have multiple applications in science and industry, such as the pharmaceutical, medical, cosmetic, food, chemistry, textile industries, and also in different processes related to biotechnology, agriculture, and the environment [1,3].

To increase the significant potential of the three natural CDs, namely α-, β-, and γ-CD, a promising line of study is represented by CD derivatives, which reinforced their potential use, being reflected in the increasing number of papers on this topic.

One particular representative of CD derivatives is represented by amphiphilic CDs, which have been designed to overcome the limitations of natural CDs for biomedical applications. Amphiphilic CDs are derivatives of natural CDs, substituted on the primary and/or the secondary face with aliphatic chains, with different lengths and structures, linked with different chemical bonds. Amphiphilic CDs are characterised by enhanced interaction with drug molecules and biological membranes, and, generally, are considered to be non-haemolytic and non-cytotoxic [4,5].

A review highlighting the important amphiphilic CD applications in the design of novel delivery systems was published by Varan et al. in 2017 [6]. The present review offers new insights into the utilisation of amphiphilic cyclodextrins by summarising the most relevant information on their structural properties, synthesis, and applications in therapy, thereby supporting the benefits of their use in the development of drug formulations with improved properties.

### 1.1. General Overview of Cyclodextrins

**Figure 1:**
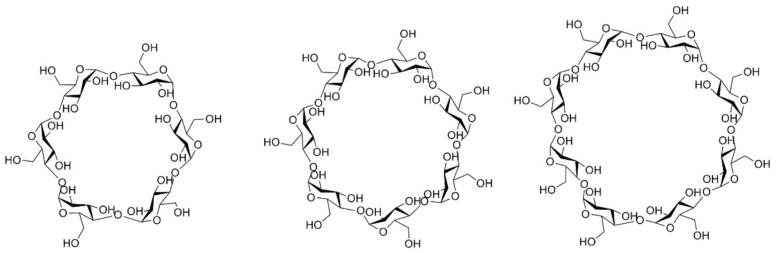
Structural formula of native cyclodextrins [14]. Data from [14], published by MDPI, 2025.

The natural CDs, α-CD, β-CD, and γ-CD are characterised by six, seven, and eight 1,4-*α*-linked glucopyranose units (Figure 1). Given the structural arrangement of the glucopyranose groups, the macromolecule takes the shape of a truncated cone or torus, having the exterior of the molecule lined by the hydroxyl functions. The secondary hydroxyl groups are situated at the wider edge, and the primary hydroxyl groups at the narrow edge. The truncated cone′s cavity is lined by the hydrogen atoms, glycosidic oxygen, and the nonbonding electron pairs of the glycosidic oxygen bridges, lending to the molecule some Lewis-base character [7]. It has been estimated that the cavities of CDs have about the same polarity as the aqueous solution of ethanol [8]. CDs have the ability to form supramolecular complexes with a large variety of drugs, without implying sophisticated chemical reactions [9,10]. Due to their biocompatibility, safety, and possibility of derivatisation, research has exponentially developed, so that currently representatives of CDs are listed in pharmacopoeias and are already used as pharmaceutical excipients, and others are available as fine chemicals [11]. CDs are included in over 40 marketed pharmaceutical products worldwide, and are found in food and cosmetic products [12,13].

### 1.2. Properties of Natural Cyclodextrins and Their Derivatives

α–, β–, and γ-CD are crystalline, homogeneous, non-hygroscopic, and water-soluble substances. The adjacent C2 and C3 –OH groups of the glucopyranose units participate in the formation of H bonds, influencing the energy of the crystal lattice in the solid state, and water solubility of the CDs. In the case of the β-CD molecule, the belt is complete, while in the α-CD structure, the hydrogen belt is incomplete, as one of the glucopyranose units is in a distorted position, and only four hydrogen bonds, of the six possible ones, are established. As a consequence, the β-CD molecule has a rigid structure and has the lowest solubility in water (1.85 g/100 mL water), and α-CD has a better water solubility (14.5 g/100 mL water). γ-CD has a non-coplanar, more flexible structure, positively influencing its water solubility (23.2 g/100 mL water) [15,16,17].

**Figure 2:**
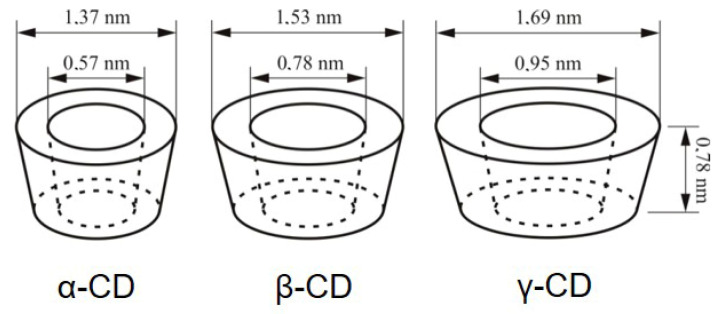
Schematic representation of natural CDs. Adapted from [16,18].

Figure 2 shows the schematic representation of the structure of natural cyclodextrins (α-, β-, and γ-CD), and Table 1 shows some of the most important structural and physicochemical characteristics of the natural CDs.

Chemically modified CDs have been obtained to surpass the limitations of natural CDs, such as water solubility, complexing capacity, and, not least, the toxicological profile [21]. Cyclodextrin derivatives are synthesised by replacing some of the primary and secondary hydroxyl groups with other functional groups [22]. The following characteristics of the hydroxyl groups need to be taken into consideration for selective modification, using moderately reactive reagents: the C2 –OH group is the most acidic, the C3 –OH group has the lowest reactivity due to steric hindrance and hydrogen bonding, and the C6 –OH group is the most nucleophilic and the most basic [23].

From the natural CDs, β-CD is the most utilised in the pharmaceutical research, having favourable characteristics, such as cavity size, suitable for inclusion of various molecules, including pharmaceutical active ingredients, complexing ability, and low cost [24,25]. β-CD has a major drawback, its low aqueous solubility, which limits its utilisation; this disadvantage is overcome by its derivatives, such as methyl, hydroxyl-propyl, and sulfobutylether derivatives, with enhanced solubility and complexing properties. The hydrophilic β-CD derivatives (e.g., randomly methylated, hydroxy-alkylated, and branched (glucosyl, manosyl) derivatives) are suitable for encapsulation of hydrophobic guest molecules, the hydrophobic β-CD derivatives (e.g., diethyl, acylated derivatives) are able to decrease and modulate the release rate of water-soluble guests, while the ionisable derivatives (e.g., anionic-β-CD) are used for enhancing the dissolution rate and the inclusion capacity [26].

Hydroxypropyl-beta-cyclodextrin (HP-*β*-CD) is one of the most used derivatives for biomedical applications, successfully overcoming the solubility and parenteral toxicity of the native β-CD [27]. The drug solubilising capacity of HP-*β*-CD was found to be, in most cases, lower than that of heptakis (2,6-di-*O*-methyl) β-CD, but, due to the lower haemolytic effect of the former upon parenteral administration, HP-*β*-CD is preferred [16].

### 1.3. Cyclodextrin Inclusion Complex Formation

The free rotation of the primary hydroxyl groups reduces the effective diametre of the cavity on their side of occurrence so that CDs are regarded as truncated cones, rather than cylinders [16].

**Figure 3:**
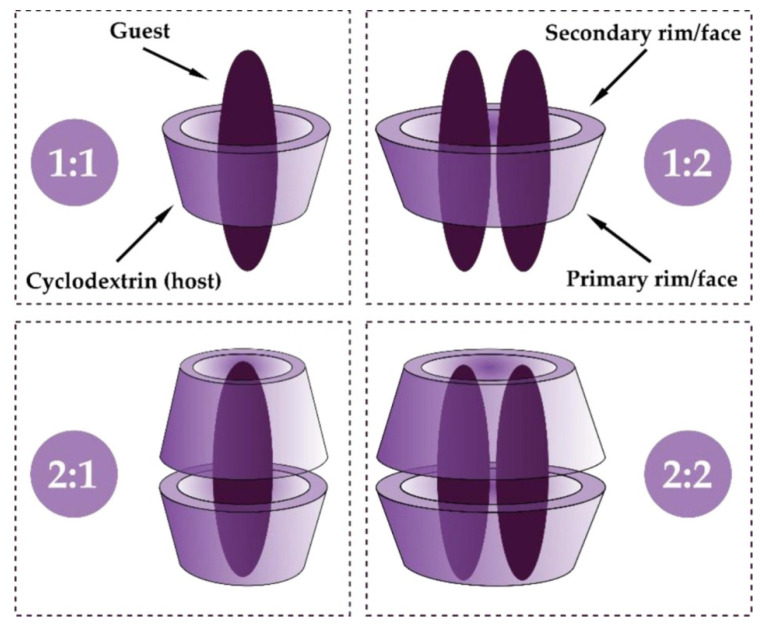
Schematic representation of the formation of an inclusion complex between CD and guest in the case of 1:1, 1:2, 2:1, and 2:2 stoichiometry (molar ratio). Data from [34], published by Elsevier, 2022.

The truncated cone of molecular size can accommodate, totally or in part, another molecule, forming an inclusion complex (IC), a process which depends on steric and thermodynamic factors (Figure 3). The molecular interaction between the guest molecule and the cyclodextrin is influenced by a variety of effects, attributed mainly to the release of inner water molecules, but also to van der Waals interactions, hydrogen bonding, and electrostatic interactions, without implying any formation or cleavage of covalent bonds [28,29,30,31].

Also, the process of inclusion complex formation is considered an entropy-driven process [10]; even so, the release of cavity-bound enthalpy-rich water molecules, situated in an energetically unfavourable polar/apolar interaction with the CD cavity, is not fully accepted by some authors as the driving force of encapsulation. These high-energy water molecules have more conformational freedom (i.e., fewer hydrogen bonds); therefore, the release process from the cavity of CDs is characterised by a negative enthalpy change [32]. Other researchers stated that the major driving forces for IC formation are considered to be van der Waals interactions and hydrophobic interactions, while electrostatic interactions and hydrogen bonding contribute to the general shape of the molecular complex [33].

Currently, CDs are considered molecular carriers for hydrophobic drug molecules by dissolving and delivering the drug molecules through the aqueous exterior of lipophilic biological membranes. CD molecules and their ICs self-assemble in aqueous solution, with the formation of nanosized aggregates that are able to solubilise the hydrophobic drugs in a micellar-like system. These aggregates are constantly being formed and disassembled; the relative amount of aggregated CD molecules increases with increasing CD concentration. In addition, the aggregation and hydration of CDs in aqueous solution might decrease the ability of dissolved CDs to form soluble ICs, thus affecting the rate of drug dissolution in aqueous CD medium [2,13,35,36].

CDs enhance the drug penetration through biological barriers, which have an aqueous exterior and a lipophilic membrane, by delivering the molecularly dissolved drug to the aqueous exterior and, thus, increasing the drug concentration gradient over the lipophilic membrane. In experiments using artificial membranes and cellular tissues, it was shown that the maximum permeation was obtained when the aqueous exterior was saturated with the drug. This particular condition enabled the maximum ability of the dissolved drug molecules to leave the aqueous exterior and to partition into the lipophilic membrane [2,35].

The dissolved state of a substance comprises a solubilised state, followed by a molecularly dissolved state, and, finally, the formation of a truly supersaturated state. In this regard, CDs were proven to induce an increased concentration of molecularly dissolved drug, with the formation of a truly supersaturated state, responsible for their positive effect on permeation. Another positive effect of CDs on permeation was proved to be their capacity to prevent the re-crystallisation of the drug from a supersaturated solution [37].

The mechanism of drug release from IC is based on simple media dilution, a change in pH, or simple dissolution of solid drug/CD complexes. They also contribute drug–protein binding affinity, drug partitioning from the complex to tissue, and competitive binding to this process [10,32].

The internal hydrophobic cavity of CDs can accommodate organic molecules, ranging from polar compounds, such as alcohols, acids, and amines, to apolar compounds, such as aliphatic and aromatic hydrocarbons, and also small inorganic anions [30].

Complexation of drugs with CDs has been widely used in pharmaceutical research to improve physical, chemical, and biopharmaceutical characteristics of drugs, such as solubility, stability, bioavailability, biological activity, and drug-excipient compatibility [38]. CDs can also offer controlled drug release profiles (immediate drug release, delayed drug release, and prolonged drug release) and reduce local irritation [39,40]. Also, CDs are used to create advanced biomaterials with applications in tissue engineering and regenerative medicine [15].

### 1.4. Toxicology Aspects

Cyclodextrins have physicochemical and biological properties similar to corresponding linear dextrins, with some differences. For example, CDs, due to their smaller size, exhibit higher osmotic properties than polysaccharides, and are more resistant to enzymatic degradation than their linear corresponding dextrins [32].

Cyclodextrins are hydrophilic molecules, with a large number of H donors and acceptors, with molecular weight ranging from 1000 to over 2000 Da, and very low octanol/water partition coefficients (log Po/w between −4.9 and −16), which hampers the permeation through lipophilic biological membranes via passive diffusion [13,32,40].

The native cyclodextrins are “Generally Recognised As Safe” (GRAS) by the European Medicines Agency [41], which allowed for the approval for use in pharmaceutical formulations. Natural α-, β-, and γ-CDs are listed in the *European Pharmacopoeia* as Alfadex, Betadex, and Gammadex, which are also 2-hydroxypropyl-*β*-CD and sulfobutyl ether β-CD sodium salt, as Hydroxypropylbetadex and Sulfobutylbetadex sodium, respectively [35].

α- and β-CDs are stable toward salivary α-amylase and pancreatic α-amylase. After oral administration, they pass through the oesophagus and stomach with negligible absorption, leave the small intestine almost intact, and undergo bacterial digestion in the cecum and the colon, with the formation of H_2_, CH_4_, and CO_2_ as metabolites. γ–CD is rapidly digested by salivary and pancreatic α-amylases and will be found in the small intestine as linear oligomers, maltose, and glucose; these products will be further transformed by bacterial digestion in the cecum and colon. The percent of non-metabolised CD in the faeces is 0.3% for α-CD, >4% for β-CD, and 0% for γ-CD [32]. The oral bioavailability of α-CD is 2–3%, approx. 0.3% for β-CD, and <1% for γ-CD; random methylated β-CD has greater bioavailability in rats [42]. Lipophilic β-CD derivatives are surface-active molecules and are, to some extent (approx. 10%), absorbed from the gastrointestinal tract [12]. The limited systemic exposure of CDs at oral administration renders them low toxicity, and, consequently, a favourable safety profile [43]. However, some studies have shown interference with lipid absorption in the gastrointestinal tract, leading to disturbances like indigestion and hepatotoxicity, with a mild increase in liver enzyme levels [15].

Parenteral administration of α-CD, β-CD, and methylated β-CDs can result in renal toxicity; therefore, they are not used in parenteral formulations [8,12,32]. Frank et al. reported the formation of acicular microcrystals and giant vacuoles in kidney tissue at subcutaneous administration of α- and β-CD [15]. The hydrophilic CD derivatives have a more favourable toxicological profile than the native CDs. For example, HP-*β*-CD is very well tolerated in humans, and after i.v. administration is eliminated through the kidneys via glomerular filtration. The lipophilic CD derivatives are not recommended for parenteral administration, being surface-active molecules and capable of binding/extracting cholesterol from the membranes. The haemolytic effect of CDs was correlated to their affinity for membrane cholesterol, and follows the order of methylated β-CDs > β-CD > HP-*β*-CD > α-CD > γ-CD > HP-*γ*-CD > Sulfobutyl-ether-*β*-CD [8].

In ocular application, α-CD and methylated β-CD caused epithelial toxicity and irritation, which were not detected for β-CD, HP-*β*-CD, or sulfobutylether-*β*-CD [41].

HP-*β*-CD and sulfobutylether-*β*-CD are approved for both oral and intravenous administration by the Food and Drug Administration. Lipophilic CD derivatives, due to their affinity for phospholipids, cholesterol, and proteins, might be useful for transmembrane absorption of drugs [15].

Cyclodextrins are biocompatible and exhibit low toxicity when used appropriately; moreover, it has been shown that CD-based biomaterials support cell viability, proliferation, and differentiation in healthy cells [15].

## 2. Amphiphilic Cyclodextrin Derivatives

Amphiphilic CDs are derivatives of natural α-, β-, and γ-CDs, modified on the primary and/or the secondary rims with aliphatic chains of varying length (C2–C18) and structure (linear/branched), linked with different chemical bonds, such as ester, ether, thiol, amide, or fluoro bonds [44,45]. Thus, by attaching single or multiple substituents, one can modulate the hydrophobic/hydrophilic balance of their structure, as well as their self-assembling properties [4], which represents one of their major advantages for biomedical applications. Moreover, the long aliphatic chains may be decorated with biological membrane-sensitive moieties, for improved cellular recognition and bioavailability [46].

### 2.1. Classification

**Figure 4:**
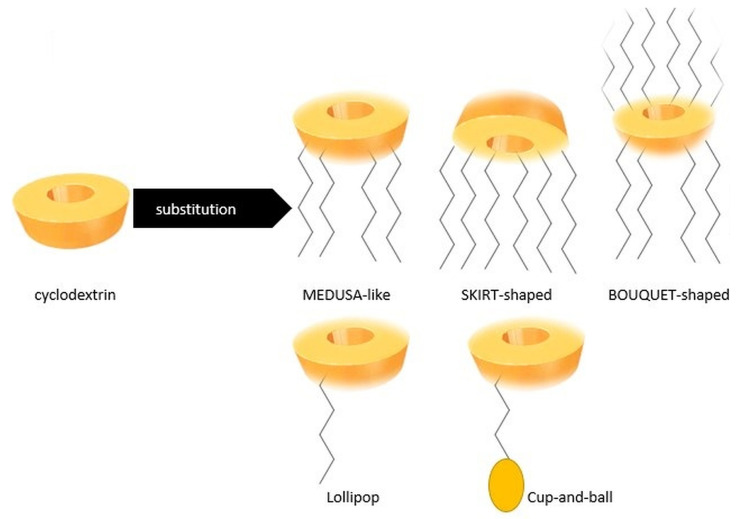
Architecture of amphiphilic CDs. Adapted from [47,48].

The substitution of the primary and/or secondary face of the torus leads to the obtention of medusa-like, skirt-shaped, and bouquet-like derivatives, which represent the three major classes of amphiphilic CDs (Figure 4) [4,47,48].

(i) The medusa-like molecules: CDs with hydrophobic substituents on the primary face.

(ii) The skirt-shaped molecules: CDs with hydrophobic substituents on the secondary face.

(iii) The bouquet-like molecules: CDs with hydrophobic or hydrophilic anchors on both faces. The medusa-like and the skirt-shaped structures can be monosubstituted and persubstituted [4].

By selective substitution with one aliphatic chain, lollipop structures and cup-and-ball structures can be obtained. The lollipop structure can be obtained by grafting only one aliphatic acid chain on the primary face of the CD. The cup-and-ball structure is formed by linking voluminous groups, like tert-butyl, to the end of the aliphatic chain of a lollipop structure, for the purpose of preventing the entry of the aliphatic chain into the cavity of the CD [49].

Amphiphilic CDs can also be differentiated as a function of the substituents inserted into the structure of the CD into non-ionic, cationic, and anionic derivatives [6].

### 2.2. Synthesis

Amphiphilic CDs can be obtained by enzymatic pathways or by chemical reactions, using amino, amido, thio, ester, ether, or fluoro bonds [4].

Chemical synthesis of amphiphilic CDs uses different approaches for narrow or wide rim modifications, as well as for different numbers of grafted anchors [50]. In case of linkage possibility, it was shown that ether bonds are the most desirable because they are less susceptible to degradation by oxidation or hydrolysis [51], while the ester bonds can be degraded in vivo by esterases [49].

In this context, some examples from the literature are given below to support the variety of chemical approaches:

2.2.1 Medusa-like CDs can be obtained using monosubstitution by grafting of single hydrophobic moieties, such as alkyl chains, fluorinated chains, cholesterol, or more complex moieties (peptidolipidyl, phospholipidyl substituents).

Polysubstituted derivatives can be obtained by adding alkyl chains (C10 to C16) or cholesterol moieties via amine, sulphur, or sulfoxide linkers.

2.2.2 The skirt-shaped structures can be obtained by esterification of the secondary hydroxyl groups (C2 and C3), using acyl donors with variable chain lengths (C4-C16).
2.2.3 Bouquet-shaped structure can be obtained by grafting hydrophobic and hydrophilic substituents on the primary and secondary faces, which leads to libraries of new compounds. For example, phospholipidyl-CDs, fluorinated-CDs, and octadecylperylene-CDs modified on both faces, with the secondary face being substituted by methyl groups, were obtained [4].

### 2.3. Advantages

Amphiphilic CDs were synthesised to improve the characteristics of natural CDs that limit their applications in the pharmaceutical field. The main advantages of amphiphilic CDs are presented below:

(i) Self-assembly in an aqueous medium, to form supramolecular structures which offer enhanced CD–drug stability upon dilution. It was shown that amphiphilic CDs can self-organise into different types of nanoassemblies (vesicles, micelles, nanorods, nanospheres, etc.) and liquid crystalline structures, without the need for an additional surfactant and/or polymer [4,30,44]. The process depends on the chemical structure, geometrical arrangement, and lipophilicity of the grafted substituents (the length, the number, and the position of hydrophobic chains), the concentration of the amphiphilic CDs, the solvent medium, and the temperature [30,52]. The aggregation process upon self-assembly might enhance the interaction of CDs with drugs, thus preventing drug efflux, which is a major disadvantage of many nanocarriers [53].

(ii) Various possibilities of functionalisation of CDs, as well as their derivatives, that enrich the possibility to obtain improved and targeted delivery systems for biomedical applications [54,55].

(iii) Improvement of the CD–drug interaction. Amphiphilic CDs have the advantage of loading the guest molecule into two distinct domains, the first being represented by the hydrophobic cavity of the CD, and the second by the lipophilic exterior (the aliphatic chains). Moreover, in the case of a nanoassembly, the entrapment efficiency is augmented by a third domain, which is the aqueous core of the nanoassembly [50,56]. The hydrophobic/hydrophilic balance and charge of moieties grafted on the CD rims determine the formation of differently shaped nanoassemblies between the drug and amphiphilic CD [57]. It was also reported that drug-loaded amphiphilic CD-based NPs prevented drug recrystallisation, thus assuring their availability for therapeutic purposes [46].

(iv) The ability to incorporate both hydrophobic and hydrophilic drugs, in conjunction with enhanced entrapment efficiency in three possible domains [58].

(v) Increased interaction with biological membranes [6]. Natural CDs are poorly transported through biological membranes, due to their hydrophilic exterior [59], while amphiphilic CDs have the ability to incorporate into phospholipid bilayer systems [4]. The bouquet-like amphiphilic CDs were developed for use as artificial transmembrane receptors (for example, amphiphilic β-CDs with *n*-dodecyl groups and *n*-hexadecyl chains on both faces) [4]. Also, the lollipop structure was shown to improve the cell targeting of the drug through liposome transport [60,61]. The affinity of amphiphilic CD for biological membranes depends on the length of the alkyl chain [53], and it can be improved by using cholesterol as a grafting moiety and by using bifunctional ligand molecules, which help form multilayers of immobilised amphiphilic CD-based vesicles [4].

(vi) Good biocompatibility and biodegradability [53,61].

(vii) A defined molecular structure, with precise size and molecular weight, essential characteristics for clinical translation and regulatory approval [62].

(viii) Improved stability as carriers for drugs, being able to release the drug progressively, as demonstrated, for example, by fluorinated amphiphilic CDs [63,64,65].

In addition, various responsive/targeting moieties can be incorporated into CD amphiphiles to enhance their applicability in therapy, such as disulfide bonds for grafting hydrophobic substituents, redox-sensitive moieties, and functional ligands, such as galactosyl and mannosyl, for cell-targeting properties [66].

### 2.4. Toxicology Aspects of Amphiphilic CD Derivatives

Although CDs are deemed Generally Recognised As Safe pharmaceutical ingredients, CD derivatives, and their inclusion complexes with drugs, demand additional findings regarding their safety and toxicity, such as the following:

(i) Data about high doses of CDs, in particular of β-CD derivatives, as gastro-intestinal and renal issues were reported on long-term use/large doses;

(ii) The safety of CDs in parenteral administration, as they have direct access to systemic circulation and, consequently, a direct impact on the organs, especially for β-CD derivatives;

(iii) The influence of the degree of substitution and the nature of substituents on the toxicological profile. For example, methylated CDs interact with cell membranes more strongly than the natural ones, which might determine membrane disruption and toxicity. Amphiphilic CDs containing aliphatic chains linked with ester bonds are considered biodegradable by esterases;

(iv) The toxicological profile of the drug can be modified through inclusion complex formation. By keeping the drug in a more water-soluble form, the absorption of the drug is increased, leading to a concentration that might exceed the therapeutic dose; the risk is of more importance in case of drugs with a narrow therapeutic index. CDs can also increase accumulation in specific tissues/organs, resulting in an increased drug toxicity in that area;

(v) Additionally, the environmental factors, such as pH, temperature, and the presence of other competing substances, might affect the pharmacokinetic profile of the included drug [43,44,67].

Research studies have pointed out that the majority of amphiphilic CDs are considered to be non-haemolytic on human blood samples and non-cytotoxic on L929 mouse fibroblast cells [4] regardless of their structure [45]. Also, amphiphilic cyclodextrins were found to be compatible with bio-membranes [61], and non-toxic against polymorphonuclear cells [44], and they showed reduced nephrotoxicity and haemolysis in the case of intravenous administration [53].

Although the Food and Drug Administration and the European Medicines Agency have both released guidance to support tolerable daily intake levels, safety margins for parenteral administration for a selection of cyclodextrins, it must be pointed out that all the toxicological data of a new CD derivative will benefit from regulatory approvals that are typically granted on a product-specific basis [67].

The toxicological data of some amphiphilic CDs discussed in this review are presented in Table 2.

## 3. Amphiphilic Cyclodextrin-Based Nanoparticles

### Preparation of Amphiphilic Cyclodextrin-Based Nanoparticles

Nanoprecipitation, the solvent evaporation method, and its modified version, the emulsification/solvent diffusion method, are generally used to prepare NPs with amphiphilic CDs [50,69].

The characteristics of the obtained NPs depend on the structural properties of amphiphilic CDs, the physicochemical properties of the drug, and the preparation technique parameters.

In the preparation of NPs, several important factors need to be taken into consideration:

(i) The cavity sizes of α–, β–, and γ-CD derivatives have no significant influence on the self-assembling properties of NPs [50].

(ii) The substitution of primary and secondary rims. A non-substituted secondary face of amphiphilic CD reduces steric hindrance and facilitates drug entrapment efficiency [68], and is favourable for host cell targeting [50]. NPs of amphiphilic CDs substituted on the primary face (for example, 6-*O*-Capro-*β*-CD, a β-CD derivative modified on the primary face with substitution of C6 linear alkyl chains) rendered a slow-release profile of the drug than β-CDC6 modified on the secondary face with C6 aliphatic chains, due to a longer maintenance of the drug in the CD cavity; this property has been observed, so far, only in the case of the NPs prepared using pre-formed inclusion complexes [68].

(iii) It is difficult to correlate the influence of the position and the number of the lipid moieties on the narrow/wide side of the rim of amphiphilic CDs with the properties of the NPs [50].

(iv) The length of the grafted aliphatic chain influences the hydrophilic–lipophilic balance, and thus the self-assembly properties. Substitution on the wide rim of β-CD with C6-C14 aliphatic chains proved to be favourable [50].

(v) Increasing the percentage of cyclodextrin and surfactant might result in particle agglomeration with a higher size and polydispersity index [54].

(vi) Alkylating the hydroxyl groups with C12 alkyl chains gives distinctive properties to amphiphilic CDs, due to the biochemical characteristics of C12 acid. Lauric acid has a faster metabolisation in the human body as compared to other saturated fatty acids, and is less accumulated in the human body; also, it was shown that lauric acid improved the ratio of total cholesterol/HDL (high-density lipoprotein) [53].

(vii) By using emulsion–evaporation methods, residual organic solvent may favour and stabilise the interaction of drug(s) with the amphiphilic CD matrix, as it may remain entangled in the NPs’ mesoporous structure. Therefore, a high recovery yield of organic solvent is required to eliminate the hypothesis of macroscopic aggregation between the drug(s) and CD [70].

(viii) In case of non-ionic amphiphilic CDs, the particle size, the polydispersity index, the physical stability of NPs, the drug loading, and the drug release are influenced by their physicochemical properties, as follows:

(a) Particle size is influenced by the substituted narrow/wide rim of the amphiphilic CD, alkyl chain length, alkyl chain nature (linear/branched), preparation technique, sterilisation method (autoclaving, filtration, gamma irradiation), and organic phase/aqueous phase ratio.

(b) The polydispersity index is influenced by the chain nature (linear/branched), sterilisation method, and organic phase/aqueous phase ratio.

(c) Physical stability is influenced by the alkyl chain length and the alkyl chain nature (linear/branched) [69].

(d) Drug loading and drug release are influenced by the physicochemical properties of the drug (aqueous solubility, molecular mass, k1:1 association constant, partition coefficient), preparation technique, and loading technique (conventional, pre-formed drug: CD complex) [69,71].

**Figure 5:**
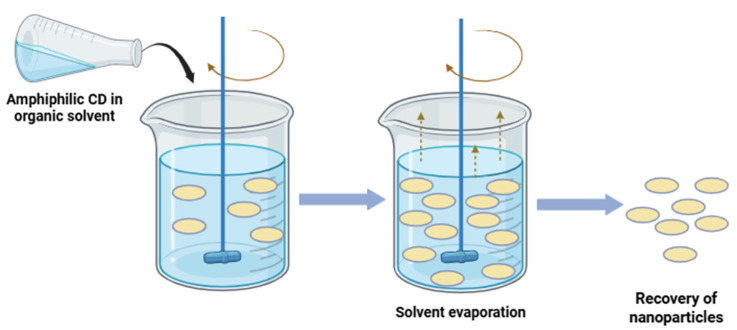
The schematic representation of the nanoprecipitation method. Adapted from [72].

Nanoprecipitation methods are generally preferred for encapsulation of lipophilic drugs [72], and can be used with or without surfactant [73]. It involves dissolution of the amphiphilic CD into a water miscible organic solvent/a mixture of water miscible organic solvents (such as ethanol, acetone, dimethyl sulfoxide, tetrahydrofuran), followed by pouring (i.e., using a silicon tube fitted with a fine tip) of the organic solution into distilled water under magnetic stirring. A colloidal suspension is formed due to the formation of nanoparticles. The evaporation of the organic solvent can be performed under reduced pressure to separate the NPs [74,75]. The schematic representation of this method is depicted in Figure 5.

For the preparation of drug-loaded nanoparticles (NPs), using the nanoprecipitation method, both the drug and the amphiphilic CD are dissolved in a water miscible organic solvent, and the rest of the method is similar to the method of preparation of blank nanoparticles [53]. The conventional nanoprecipitation method was further modified by using pre-formed inclusion complexes instead of the drug, which proved to be an effective method to enhance drug loading capacity and influence in vitro drug release. The drug entrapped in the CD cavity is released from the NPs in a time-dependent mechanism, involving dissolution upon dilution and competitive displacement of the drug from the CD cavity by medium constituents.

Regarding the preparation of drug-loaded NPs, particular attention must be given to the so-called burst effect, correlated to the fraction of drug located at or near the surface of the NPs, which immediately dissolves and diffuses into the dissolution medium [68]. For example, a series of amphiphilic CD derivatives, namely, β-CDC6, a β-CD derivative modified on the secondary face with 6C aliphatic esters, and 6-*N*-Capro-*β*-CD, a β-CD derivative modified on the primary face with 6C aliphatic amides, were employed for the preparation of NPs, using pre-formed drug/amphiphilic CD inclusion complex. The obtained NPs were characterised by high drug loading capacity and a reduced burst effect during the drug release process [45].

Baâzaoui et al. have successfully prepared NPs employing the nanoprecipitation method and the dispersion by ultrasound method, using amphiphilic β-CD derivatives, substituted at the primary face, with either one or seven amino-alkyl chains (n-butyl or n-dodecyl). Although both methods yielded stable NPs, their physicochemical properties were very different, depending on the preparation method: large (>500 nm) and polydisperse NPs for the ultrasound method, and small NPs (100–200 nm) with a narrow particle distribution when using the nanoprecipitation method. The experiment showed that the dispersion of NPs was influenced by CD concentration, since it was obtained at a concentration larger than the solubility; the chemical structure had no important influence. Also, the concentration domain necessary for the formation of NPs was quite narrow for dodecyl-chained derivatives, especially when using the ultrasound method. Although the amphiphilic CD derivatives were essentially neutral and insoluble in water, the obtained NPs were characterised by positive charge, due to the weak protonation of the amino groups. The cationic character may be an advantage when adsorption or deposition at the surface of negatively charged materials, such as hair, skin, and fabrics, is required [52].

Cho et al. obtained a ladle-type amphiphilic 2-*O*-mono-lauryl β-CD and investigated its potential to form NPs, using the nanoprecipitation method. Uni- or multilamellar vesicles with diameters of 60-250 nm were obtained. In this regard, the authors proposed that the self-assembling vesicular structure could be applied for drug delivery [61].

Heptakis-6-*O*-hexanoyl-*β*-CD (CDOC6), heptakis-6-*O*-lauroyl-*β*-CD (CDOC12), and heptakis-(6-deoxy-6-hexylthio)-*β*-CD (CDSC6) were obtained by linking the aliphatic chains via ester or thioether bonding and were further used for nanoparticle preparation, using the nanoprecipitation method. The assessment of physicochemical properties of obtained NPs revealed the influence of the type of linking structure and the length of the alkyl chain on the properties of the obtained NPs.

The thioether linkage in CDSC6, due to its higher lipophilicity, enhanced molecular attraction, resulting in larger particles over 200 nm in diametre, while the angled ester bond in CDOC6 and CDOC12 derivatives sterically hindered molecule accumulation. Among ester-bonded derivatives, the larger particles were observed for the CDOC12 derivative, attributable to its longer alkyl chain. The NP size for CDOC12 and CDSC6 derivatives was similar, highlighting the significant role of linking structure over the length of the aliphatic chain [76].

Li and co-workers obtained amphiphilic multi-charged cyclodextrins and vitamin K co-assemblies, with synergistic coagulant activity, using the film hydration method. The amphiphilic CDs and vitamin K were dissolved in chloroform, followed by evaporation of chloroform using a rotary evaporator; a thin lipid film was formed. The lipid film was added to water, stirred for 0.5 h at 55 °C, and sonicated for another 0.5 h at the same temperature. The obtained solution was dialysed for 3 h to remove the unloaded vitamin K. As amphiphilic CDs, a series of 6–deoxy-*β*- and 6–deoxy-γ-CD derivatives was used, substituted at C6 with 1-butyl-imidazole or 1-octyl-imidazole. The method was proven to provide a promising candidate for designing a clinical anti-heparin coagulant. Moreover, the multi-charged character of amphiphilic cyclodextrin was beneficial, offering multivalent bonding and neutralising effect of heparin, rather than protamine in plasma [77].

Stancanelli et al. prepared nanoaggregates of non-ionic amphiphilic CD, namely [(2-oligo-ethylenoxide-6-hexylthio)-*β*-CyD, SC6OH] and genistein, using the emulsification–diffusion method, a method usually leading to the obtention of aggregates with low colloidal stability. Despite this, the obtained NPs showed a good colloidal stability. The residual solvent determined the formation of supplementary nanodomains in which host/guest complexes were included. The results prompted the authors to reconsider the role of residual organic solvent in designing drug delivery systems [78].

## 4. The Role of Amphiphilic Cyclodextrins in Enhancing the Therapeutic Efficacy of Drugs

### 4.1. Amphiphilic Cyclodextrins in Anticancer Drug Delivery

Cancer is one of the leading causes of death in the world. The replacement of conventional therapy, subjected to many therapeutic failures and severe adverse effects, is one of the primary goals in the field of related biomedical research. An ideal anticancer drug carrier is primarily expected to deliver an adequate drug concentration at the tumour site, with minimum secondary effects and complications. The other desired characteristics of an anticancer drug delivery system are biocompatibility and biodegradability, solubility improvement of the drug, a controlled and sustained release of cargo, and protection from enzymatic or environmental degradation [79].

**Figure 6:**
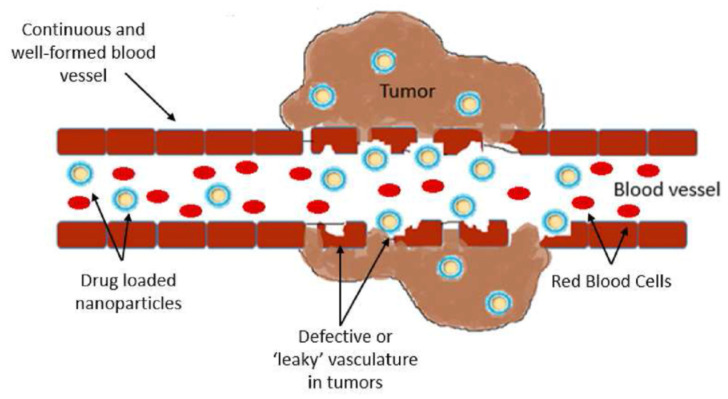
The schematic representation of the EPR. Data from [84], published by Elsevier, 2023.

Tumour cells have specific characteristics in their intracellular environment, such as decreased pH, increased glutathione (GSH), overexpression of certain enzymes, and increased oxygen species [80]. The enhanced permeability and retention effect (EPR) occurs in solid tumour tissues and is characterised by a defective architecture of blood vessels [81] and excessive production of vascular mediators [82]. The enhanced permeability and retention effect has now been accepted as one of the universal pathophysiological characteristics of solid tumours, and is considered a fundamental principle for effective nanomedicine applied in cancer [83]. The schematic representation of the EPR is presented in Figure 6.

The major advantages promoted by drug loading into molecular carriers are overcoming the body barriers, such as degradation in the gastrointestinal tract, and the drug disadvantages, such as poor water solubility, poor membrane permeability, and low stability [79].

In this light, nanoparticle-based delivery systems have been extensively exploited in controlled drug delivery due to their advantageous properties, strongly influencing the therapeutic efficacy of the carried drug. The particle size, surface charge, drug loading, and type and material of NPs offer multiple ways to obtain modern therapeutic delivery systems for different purposes. In cancer therapy, NPs have proved to overcome the traditional chemotherapy limitations, being able to improve drug selectivity, reduce side effects, and ameliorate the resistance to chemotherapy [84,85,86]. Moreover, some NPs were designed as tumour microenvironment-responsive drug self-delivery systems, with great potential for treating malignant tumours with multidrug resistance [80].

Cyclodextrins have served as excellent materials for nanostructured therapeutic platforms, leading to new cancer therapy alternatives [87], and amphiphilic CDs are promising drug carriers thanks to their own properties in addition to the characteristics of NPs.

In this section, we will highlight the role of the architecture of CD derivatives and of modifications in preparation methods for obtaining amphiphilic CD-based NPs loaded with anticancer drugs.

Çirpanli et al. emphasised the role of substitution of the narrow/wide rim of the amphiphilic CD molecule, as well as the utilisation of pre-formed drug: CD inclusion complexes in improving solubility, stability, and biological activity of anticancer drug camptothecin. The drug exists in two forms: the active lactone form at a pH below 5, and the inactive carboxylate at a basic pH. The preparation of NPs was performed using the nanoprecipitation method and pre-formed inclusion complexes between camptothecin and β-CD derivatives. As CD derivatives, 6-*O*-Capro-*β*-CD amphiphilic derivative substituted on the primary face with C6 linear alkyl chains via ester bonds, and β-CDC6 amphiphilic derivative, substituted on the secondary face with C6 aliphatic esters, were used. The camptothecin/CD NPs increased the stability of the anticancer drug against hydrolysis and were able to maintain the drug in its lactone active form. The substitution on the primary side, as seen in the case of amphiphilic 6-*O*-Capro-*β*-CD, was beneficial in terms of higher drug loading efficiency, a slower release profile than β-CDC6 derivative modified on the secondary face, and anticancer efficiency on MCF-7 breast adenocarcinoma cells. The authors proposed the two CD derivatives, and especially 6-*O*-Capro-*β*-CD-based NPs, for further in vivo experiments, which are safe, effective, and have a particle size suitable for parenteral administration [68].

In the above-mentioned study, Çirpanli et al. also revealed the advantages of amphiphilic CDs regarding the loading capacity, enhanced tumour retention and biocompatibility as compared to polymeric NPs based on poly(lactide-co-glycolide) (PLGA) and poly-ε-caprolactone (PCL). The loading capacity of camptothecin in amphiphilic CDs (β-CDC6 and 6-*O*-Capro-*β*-CD) was higher compared to PLGA and PCL NPs. (The polymeric NPs with camptothecin were prepared using camptothecin:HP-*β*-CD ICs.) Camptothecin encapsulated in amphiphilic β-CDs (6-*O*-Capro-β-CD, in particular) showed superior cytotoxic effects toward MCF-7 cell lines, compared to PLGA and PCL/camptothecin NPs, and low cytotoxicity on L929 fibroblasts [68]. These results were further correlated by the same authors in another study, with an in vivo rat model of 9L gliosarcoma cells, showing a longer surviving time in the case of camptothecin-loaded 6-*O*-Capro-*β*-CD NPs as compared to camptothecin administered via β-CDC6/PLGA/PCL NPs [88].

The effect of substitution on the primary and secondary face of β-CD was also assessed by Erdoğar et al. [54] for obtaining paclitaxel-loaded folate-conjugated amphiphilic CD NPs. The amphiphilic CD derivatives were designed to enable active targeting to folate-positive human breast cancer cell lines. Folate conjugated amphiphilic β-CD derivatives were obtained by modifying the secondary (FCD-1)/primary (FCD-2) face with C6 linear alkyl chains by esterification. The folate residue was attached by click chemistry on the substituted face at the end of the C6 linker chain. The FCD-1 derivative contained 14 alkyl residues, while the FCD-2 derivative contained 7 alkyl residues, resulting in higher lipophilicity and molecular weight for the first one. The preparation of the paclitaxel-loaded NPs was assessed using the nanoprecipitation technique.

The secondary face-substituted (FCD-1) NPs encapsulated a higher amount of paclitaxel, and, subsequently, displayed a slower release of drug; the results were in good agreement with cell culture studies. This was expected because FCD-1 NPs, with a more hydrophobic structure depending on a large number of aliphatic chains, exerted a stronger interaction with paclitaxel. The paclitaxel-loaded NPs maintained the physical stability of the drug in aqueous solution for one month and avoided re-crystallisation, one of the major causes of undesired side effects.

Blank FCD-1 NPs had a higher uptake by folate receptor-expressing breast cancer cell lines, T-47D and ZR-75-1, and also paclitaxel-loaded FCD-1 ones, as a result of smaller particle size, neutral surface charge, and higher encapsulation efficiency. On T-47D cells, both types of loaded NPs displayed comparative anticancer efficacy to that of paclitaxel solution alone, while in the case of ZR-75-1, they increased the anticancer effect of paclitaxel. Moreover, paclitaxel-loaded FCD-1 NPs were effective at a lower concentration than paclitaxel FCD-2 NPs, making them suitable candidates for safe and effective delivery of paclitaxel with a folate-dependent mechanism [54].

The modified ethanol injection method was used to prepare tamoxifen citrate-loaded nanovesicles based on amphiphilic β-CDC12. First, amphiphilic CD was obtained by substituting a C12 alkyl chain to β-CD in a single-step method; a bouquet-type of amphiphilic CD was obtained. Then, blank nanovesicles were obtained, where surfactant was dissolved in water, and amphiphilic CD was dissolved in ethanol. For the preparation of nanovesicles loaded with tamoxifen, the authors did not use a pre-formed inclusion complex, with both tamoxifen and amphiphilic CD being dissolved in the organic phase (ethanol). This conventional loading method was successfully employed; the amphiphilic CDs self-assembled in aqueous medium, forming perfectly spherical nanovesicles with a size less than 200 nm. Tamoxifen was encapsulated into the nanosystem, forming a stable complex, from which it was released slowly, following Fickian diffusion. The amphiphilic CD exhibited significantly lower haemolytic potential as compared to parent β-CD, making it a suitable carrier for parenteral delivery. Cytotoxicity studies on MCF-7 cancer cells revealed that amphiphilic CD was not cytotoxic, and enhanced the cytotoxicity of tamoxifen, even at low concentrations, as the carrier was able to assure a slow release of tamoxifen. Moreover, the IC_50_ of tamoxifen was significantly reduced after encapsulation in nanovesicles. The in vivo experiments in mice revealed that blank amphiphilic CD was well-tolerated when administered intravenously. The in vivo pharmacokinetic studies on rats demonstrated an extended retention time of tamoxifen in plasma when encapsulated in nanovesicles. The study successfully delivered a one-step synthesis for randomly alkylated amphiphilic CDs, and the obtention of a safe, viable, new delivery system for tamoxifen, for parenteral delivery [53].

Nanoparticles of the amphiphilic β-CD derivative, modified on the secondary face with 6C aliphatic esters (β-CDC6), were obtained using the nanoprecipitation technique, in the presence and absence of Miglyol 812. In the presence of surfactant, nanocapsules were obtained, while in the absence of Miglyol 812, nanospheres were obtained. Then, loaded NPs were obtained, using pre-formed tamoxifen: β-CDC6 complexes. Blank and loaded NPs were characterised by particle size between 280 and 330 nm, and the polydispersity indices were below 0.1 for all formulations. Tamoxifen citrate-loaded nanospheres showed a significantly faster release profile than the corresponding nanocapsules, and were proposed for further investigations to target solid tumours following i.v. injection. The biological study revealed that the antiestrogenic activity of tamoxifen citrate depended on the concentration of 17-β estradiol, since NPs loaded with the drug did not inhibit the estradiol-mediated luciferase gene expression in MELN cells [89].

In addition to the above-described delivery system, Memisoglu-Bilensoy and colleagues have synthesised NPs based on similar amphiphilic β-CDC6, aiming to incorporate a significant amount of tamoxifen citrate and to achieve a relatively slower in vitro release profile of tamoxifen citrate. The NPs were prepared by the nanoprecipitation method and were loaded conventionally/using pre-formed inclusion complexes of tamoxifen citrate and β-CD6 (1:1 molar ratio). The findings highlighted the importance of using pre-formed drug: CD inclusion complex in the preparation of NPs, resulting in the formation of stable NPs of high drug loading capacity, and a reduction in burst effect during the drug release process. Moreover, it was shown that using an additional drug solution in the organic phase for obtaining highly loaded NPs led to drug loss during preparation. Pre-loaded formulations showed a significantly slower release profile extended up to 6 h, while conventionally loaded NPs displayed rapid and complete release within 1 h [44].

Amphiphilic CDs were tested as an alternative formulation approach to current commercial products containing Cremophor EL as a solubiliser of paclitaxel [45]. Cremophor EL exerts a range of effects, some of them with important clinical implications, such as severe anaphylactoid hypersensitivity reactions, aggregation of erythrocytes, and peripheral neuropathy. Moreover, it was shown that it has a dose-independent pharmacokinetic profile, and a clearance highly influenced by duration of the infusion, and can modify the toxicological profile of certain anticancer drugs [90]. Paclitaxel in commercial products formulated with cremophor has a limited bioavailability, important side effects associated with cremophor, and tends to precipitate in aqueous medium. In this regard, Bilensoy et al. aimed to prepare an alternative formulation for injectable paclitaxel, Cremophor EL-free, to reduce paclitaxel side effects and increase the therapeutic efficiency. Paclitaxel-loaded/blank NPs were prepared using the nanoprecipitation technique. Pre-formed inclusion complex of paclitaxel: 6-*O*-Capro-*β*-CD (β-CD derivative modified on the primary face with 6C aliphatic ester) was used for the preparation of NPs. By comparing the safety of blank nanoparticles against commercial vehicle cremophor/ethanol (50:50 *v*/*v*) by haemolysis tests and cytotoxicity experiments, data revealed that blank NPs caused significantly less haemolysis and were not toxic against L929 mouse fibroblast cells. Inclusion of paclitaxel into CD-based NPs increased its stability, with recrystallisation of paclitaxel not being observed during a one-month test. Anticancer efficiency of paclitaxel NPs tested on MCF-7 breast cancer cells was slightly higher than the paclitaxel solution in the cremophor/ethanol mixture. These findings motivated the authors to advance amphiphilic CD NPs loaded with paclitaxel as a promising alternative for the injectable formulation [45].

In another study conducted by Ercan et al., blank polycationic amphiphilic β-CD (PC βCDC6) nanoparticles were synthesised using the nanoprecipitation technique and their cytotoxic effect was evaluated on hepatic tumour cell line HepG2. PC βCDC6 presented a belt of 7 primary amino groups on the primary face of the CD, which were protonated at physiological pH, and 14 hexanoyl chain modules on the secondary face, rendering a reversed facial amphiphile structure. The blank CD-based polycationic nanoparticulate delivery system proved remarkable antiproliferative properties on HepG2 cells, and, due to their strong positive charge, might have an innate ability to penetrate and accumulate at the liver tumour site. The blank Nps were able to trigger apoptosis, decrease the amount of cholesterol, and change the morphology and viability of cancer cells. Also, the potential to overcome the drug resistance of HepG2 cells, due to alleviation of the activity of p-glycoprotein, was revealed. These evaluations encouraged further investigation of the PC βCDC6 as a convenient carrier for targeted chemotherapeutic agent administration in patients with hepatocellular carcinoma [91].

Amphiphilic CDs have shown potential application for dual chemotherapeutic and photodynamic drug delivery systems in nanomedicine. Moreover, their surface may be modified with receptor targeting groups, suggesting the in vivo translational potential of this type of nanocarrier.

In this context, Conte et al. obtained non-ionic self-assembling amphiphilic β-CD derivatives based on heptakis (2-oligo(ethyleneoxide)-6-hexadecylthio)-*β*-CD. The resulting CD derivatives were used to encapsulate zinc (II)–phthalocyanine for photodynamic therapy of cancer cells. The encapsulated zinc (II)–phthalocyanine was able to avoid aggregation and to remain active in monomeric form, enabling it to produce singlet oxygen upon irradiation, leading to a photodynamic therapeutic effect on HeLa cancer cells, comparable to that of the free drug. The results advance the potential of using this type of NPs in in vivo experiments [92].

Continuing their previous research, Conte et al. obtained nanoassemblies of non-ionic amphiphilic CDs based on heptakis (2-oligo(ethyleneoxide)-6-hexadecylthio)-*β*-CD (SC16OH) for dual delivery of docetaxel and zinc (II)–phthalocyanine. The architecture of molecular assembly revealed a selective interaction of docetaxel with hydrophobic SC16 groups of two adjacent CDs, while the interaction of photosensitiser with amphiphilic CD was established through the oligo-ethylenglycol and the thioalkyl moieties, thus being entrapped both in hydrophobic and hydrophilic side chains of CD. Moreover, the nanoassembly allowed for a monomeric inclusion of photosensitiser, with a low tendency to aggregate. The dissolution profile of NPs revealed a sustained release of the entrapped drugs. The haemolytic activity was investigated, showing a very low haemolytic activity (≈10%) for both unloaded and loaded NPs. In HeLa cells, zinc (II)–phthalocyanine photodamage enhanced the susceptibility to docetaxel cytotoxic effects. Taking all these together, the in vivo translational potential of this type of nanocarrier was proposed [70].

### 4.2. Folate-Targeted Amphiphilic Cyclodextrin Nanosystems

Folic acid (pteroyl-L-glutamic acid, vitamin B9) is indispensable for DNA replication and repair, and RNA synthesis. The expression of folate receptors in normal cells is low and is limited to cells involved in embryonic development or folate reabsorption. The overexpression of folate receptors is found in several pathologies, such as inflammatory diseases, and a variety of cancers, including breast, kidney, colorectal, brain, and ovarian cancers [93,94].

Folate uptake by cells is mediated by folate receptor in granulocytes, monocytes, and especially in macrophages (myeloid immune cells), which constitute the reticuloendothelial system (RES) and infiltrate the tumour microenvironment. As a consequence, both cancer cells and immune cells infiltrating the tumour tissue have higher folate requirements for DNA synthesis and repair mechanisms [95].

These findings rendered the overexpression of folate receptors as a therapeutic target for improved drug delivery, using folate-decorated delivery systems, which proved the following advantages: higher tumour permeability, higher stability in acidic/alkaline environments, and relatively low toxicity. The specific affinity of folic acid to folate receptor promotes the internalisation of folate-targeted NPs into the cytoplasm through receptor-mediated endocytosis, followed by the release of NPs in the cytoplasm [94]. However, the release of the drug from the endosomes is at a low rate, therefore representing one of the major limitations of gene therapy, which needs to be addressed [96].

**Figure 7:**
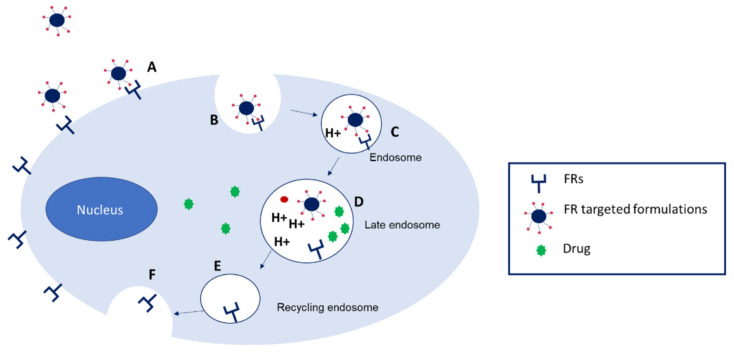
Schematic representation of internalisation of FR-targeted delivery systems. The folic acid/ another ligand binds to folate receptor (A), resulting in an invagination of the plasma membrane (B). The folate receptor-ligand conjugate is enclosed in endosome (C), followed by the release of the ligand (D) and the drug into the cells. Finally, the recycling of the folate receptor to the cell surface, occurs(E, F). Data from [93], published by MDPI, 2021.

The schematic representation of FR targeted delivery systems is depicted in Figure 7 [93].

It is possible to perform active targeting with charged/non-ionic amphiphilic CDs, as presented below.

The research group of Aranda et al. developed polycationic amphiphilic CD (paCD)—pDNA (luciferase encoding plasmid DNA) nanocomplexes (paCD-pDNA), with improved tumour cell-discriminating abilities for cancer gene therapy, using post-decoration of pre-formed CDplexes with folic acid. For the formation of CDplexes, a β-CD derivative having a tetradecacationic structure incorporating 14 primary amino groups and 7 thioureido groups at the primary face, and 14 hexanoyl chains at the secondary face, with positive surface potential was used. This type of paCD has been previously shown to promote transfection in several cell lines and to complex and compact plasmid DNA into CDplexes. Post-decoration of CDplexes with folic acid was based on electrostatic interaction with the vitamin, to form ternary paCD:pDNA:FA nanocomplexes. Folic-CDplexes proved to be efficient protectors of pDNA by forming monodispersed and stable NPs, and promoted transfection in HeLa cells, with no associated toxicity. In this respect, it is well known that polycationic macromolecules might cause a destabilisation of the cell membrane, leading to cell lysis. Moreover, in a mouse model, when administered intravenously, these NPs assured high transfection levels in the lung, and especially in the liver, compared to non-targeted CDplexes. The formulation was found to be suitable, in terms of composition and translation into large-scale production, for obtaining an alternative platform to viral vectors for gene therapy [96].

Zagami et al. obtained a ternary nanoassembly composed of non-ionic amphiphilic β-CD incorporating pheophorbide as a phototherapeutic agent and an adamantanyl–folic acid conjugate to target tumour cells overexpressing alpha-folate receptor FR-α(+). The determination of the morphology of NPs indicated the presence of near-spherical assemblies of approximately 280 nm in size. The ternary nanoassembly was successfully internalised into human breast MCF-7 cancer cells (overexpressing alpha-folate receptors) as compared to human prostate carcinoma PC3 cells (with low levels of folate alpha receptors). This was also supported by a spectroscopic study showing that folic acid protruded out of amphiphilic CD rims, for recognition with alpha-folate receptors. Moreover, the ternary nanoassembly showed an improved phototoxicity in folate receptor FR-α(+) MCF-7 cells versus the nanoassembly designed without the adamantanyl–folic acid conjugate as a targeting unit; the ternary assembly favours the monomeric form of pheophorbide, responsible for generating singlet oxygen necessary for ensuring phototherapeutic action. The ternary complexes remained stable for 2 weeks in aqueous solution. The experiments in environments mimicking physiological conditions indicated the stability of nanoassembly in the presence of serum proteins and a sustained release of pheophorbide, thus advancing the possibility of maintaining a high integrity of assembly within 24 h (average time to reach the target sites) [97].

Small interfering RNA (siRNA) is a noncoding RNA (20–30 nucleotides) that regulates genes and genomes by RNA interference (RNAi), inducing gene silencing [98,99]. The siRNA-based delivery systems have gained attention as a therapeutic strategy for the treatment of several pathologies, especially against those difficult to treat with drugs [100]. Moreover, some siRNA drugs have already been approved for clinical use by the Food and Drug Administration, such as Patisiran and Givosiran, used to treat hereditary transthyretin-mediated amyloidosis and acute hepatic porphyria, respectively [101]. siRNA possesses a short in vivo half-life, as it is rapidly degraded by nucleases in the plasma and cleared by the kidneys; therefore, the therapeutic properties of this molecule are difficult to control [102]. Moreover, for therapeutic purposes, the large anionic molecule of siRNA requires a targeted delivery, a low stimulation of the immune system, and serum stability. This can be addressed by using several carriers, including amphiphilic CDs [103].

In this context, Evans et al. have successfully obtained folate-targeted amphiphilic CD-based NPs formulated for siRNA delivery to prostate cancer cells. The amphiphilic SC12CDclickpropylamine CD, with cationic groups on the secondary face and C12 on the primary face, was used to complex siRNA. Then, DSPE-PEG5000-folate and the GALA peptide were inserted into pre-formed CD:siRNA complex in order to enhance the targeting potential and the endosomal escape of the drug. GALA is a 30-amino-acid peptide, derived from viral proteins, that enhances endosomal release following its uptake. The NPs displayed favourable physicochemical properties, maintaining a particle size less than 200 nm over 24 h, and the CD and PEG were able to protect siRNA from serum nucleases degradation for up to 8 h. The uptake of NPs, assessed in two prostate cancer cells, PC3 and LNCaP, with different pathways of folate uptake, was significantly increased, as compared to folate-untargeted NPs, used as a control; also, a significant reduction in the therapeutic targets and protein levels was revealed. The addition of GALA significantly increased the level of knockdown. The results endorsed the effectiveness of using pre-formed complexes for the preparation of NPs, and also of including DSPE-PEG5000 and GALA, in the formulation of folate-targeted CD vector [103].

Amphiphilic cationic CD-based NPs modified with PEGylated folate were developed for co-delivery of docetaxel and siRNA (against the RE1A, a subunit of nuclear factor-κB), in a colorectal cancer model. It is well known that docetaxel is less effective in colorectal cancer, and the role of nuclear factor-κB in the maintenance of docetaxel resistance; therefore, the combination aimed to enhance the drug efficacy via targeting folate receptors and RE1A. The co-formulation, CD:DTX:siRNA:DSPE-PEG2000-FA, demonstrated favourable physicochemical properties, drug loading efficiency, and a higher release in an acidic environment (thus facilitating the release in the endosomes, a pH of 5.5–6.5), which are compliant with the requirements for improved pharmacokinetic properties. In addition, the co-formulation achieved cell-specific uptake, significantly higher compared to the non-targeted counterpart, and improved the apoptotic effect of docetaxel with a reduction in the expression of Re1A mRNA and protein. As a consequence, a remarkable retardation of the growth of colorectal cancer in mice, without causing significant toxicity, was observed [104].

### 4.3. Cholesterol-Targeted Amphiphilic Cyclodextrin Nanosystems

Researchers have found that lipids sustain rapid tumour growth and also contribute to the resistance of tumours to chemotherapy. The lipid metabolism is triggered by the tumour, providing high levels of ATP (adenosine triphosphate), growth factors, and cytokines. In particular, the change in cholesterol metabolism is considered the typical characteristic of lipid metabolism reprogramming in cancer [80]. Cholesterol contributes to cancer development by modulating immune response, ferroptosis, autophagy, and the DNA damage response [105]. Cholesterol interacts with various well-known cancer-related signalling pathways, including the hedgehog, Wnt, and p53 pathways. Moreover, cholesterol metabolites, such as cholesterol esters, oxysterols, and bile acids, as well as their biosynthetic intermediaries such as squalene and isoprenoids, have been proven to be involved in cancer progression [106].

Cholesterol is a major component of cellular membranes, subsequently determining the biophysical parameters of these membranes. Cancer cells have a different membrane lipid composition than healthy cells, with the number of cholesterol domains being very high, especially in drug-resistant cancer cells.

**Figure 8:**
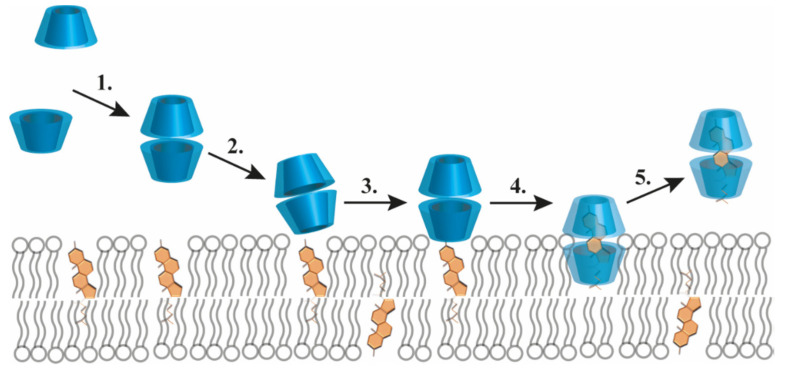
Schematic representation of CD (represented in blue)-mediated cholesterol (represented in brown) extraction from cellular membrane. Data from [108], published by MDPI, 2022.

Natural CDs, due to their hydrophilic nature, have a low potential to permeate cell membranes, but some of their derivatives, which are more hydrophobic, have been reported to extract cholesterol from cell membranes, leading to cellular apoptosis. Cholesterol extraction by CDs results in substantial alterations of membrane properties, such as increased fluidity, hydration, and elasticity, and decreased lipid order, bilayer thickness, and dipole potential [107,108]. In this regard, CDs demonstrated their potential as therapeutic agents in neurodegenerative disorders, like Niemann–Pick type C disease and Alzheimer’s disease [18]. The schematic representation of CD-mediated cholesterol extraction from the cellular membrane is depicted in Figure 8 [109].

Varan et al. prepared NPs from non-ionic (6-*O*-capro-*β*-CD) or poly-cationic amphiphilic CD (PC βCDC6) and comparatively evaluated them for apoptotic and cytotoxic effects on tumour cell models. Blank NPs exerted cytotoxic effects on a variety of cancer cells (MCF-7, HeLa, HepG2, MB49), and did not affect healthy cells (L929, G/G). Moreover, blank 6-*O*-capro-*β*-CD and blank PC βCDC6 derivatives were found to have an intrinsic efficiency on cell number and membrane integrity of MCF-7 cells in apoptosis studies. The apoptosis of cancer cells was attributed to the cholesterol extraction ability of these CDs [5].

### 4.4. Amphiphilic Cyclodextrins for Gene Delivery

Nowadays, gene therapy represents a modern domain of research and is extremely active, as it is designed to target the source of disease, and not just the symptoms [47]. Gene therapy, which implies the introduction of specific genetic material into cells, is used to increase the expression of the transferred gene to silence the expression of the disease-causing gene or to remove/replace/insert a gene with its functioning version [109,110]. In this respect, gene delivery systems must be designed to protect the genetic material from premature degradation in the systemic blood stream, and to efficiently transfer the therapeutic genes to target cells [96].

Cyclodextrins have been explored as carriers for several types of gene therapy, as they are often one of multiple components in gene delivery systems. Natural CDs do not have the innate potential to complex the genetic material, and have limited transfection ability. Therefore, the usefulness of the CDs in gene delivery can be exploited by modulating their physicochemical properties, such as charge density, hydrophilic–hydrophobic ratio, by adding functional groups, and spacer length. Cationic CDs allow for electrostatic interaction with nucleic acids having negative charges, such as plasmid DNA (pDNA), micro RNA, small interfering RNA (siRNA), messenger RNA (mRNA), and even CRISPR-Cas9 genome editing machinery, with formation of so-called “polyplexes” [47,111]. It was also shown that cationic CDs, with their overall positive surface charge, interact electrostatically with negatively charged cell membrane components (proteoglycans, glycosaminoglycans), thus enhancing cellular uptake and transfection efficiency [112]. Moreover, amphiphilic CDs revealed intrinsic advantages in gene condensation and delivery, such as avoiding degradation of genes, improvement of membrane permeation, and targeted delivery, serving as a class of non-viral vectors [62].

Geng et al. reviewed a series of macrocyclic molecules used as gene carriers, among them amphiphilic CDs modified with cationic groups, and amphiphilic CDs with saccharide targeting groups, highlighting important conclusions. In case of the amphiphilic cationic CDs, the following structural characteristics were favourable: (a) the β-CD scaffold, due to complexation properties for cholesterol, (b) in a homologue series, the branched structure versus the linear one was more favourable, (d) increasing the number of ethyleneimine segments per branch, and incorporating thiourea segments enhanced the transfection capacities, (e) the hydrophilic/hydrophobic ratio was important to efficient self-assembly in the presence of DNA, (f) the internalisation was reached through clathrin- and caveolin-dependent endocytosis, with the latter being dominant for transfection; moreover, in vivo transfection occurred mainly in the liver and partially in the lungs, proposing these vectors for cytokine-based hepatocarcinoma. In case of amphiphilic CDs with saccharide targeting groups, functionalisation with mannose, aminoglucoside, galactosyl, and lactose suggested promising perspectives [62].

Kont et al. [113] studied a series of β- and γ-cationic amphiphilic CDs aiming to determine the structure–activity relationship for enhanced siRNA delivery.

The following remarks were concluded: (a) the presence, at position C2, of a primary amine in both amphiphilic β- and γ-CD derivatives resulted in superior gene knockdown effect compared to tertiary amines; (b) the presence of two primary amine groups at C2 and C3 enhanced the level of gene knockdown; (c) primary amines seems to modulate proton influx into the endosome, leading to osmotic swelling and disruption; (d) modification of the secondary rim of γ-CD with two sets of primary amine groups using a thiopropyl linker, as compared to triazole linker, is more favourable in achieving gene knockdown, regardless of the dose; (e) co-formulation with PEGylated CDs is beneficial for in vivo administration, due to modulation effect of cation charge [113].

#### 4.4.1. Amphiphilic Cationic CDs in Gene Delivery

Amphiphilic cationic CDs, which self-assemble into NPs, have been shown to enhance membrane permeability, promoting intracellular delivery of siRNA [114] and of pDNA [115] for modulation of gene expression.

siRNA presents the potential of specific silencing of target genes associated with neurodegenerative and cancer disease progression. A series of barriers for siRNA delivery have been identified, which impairs the therapeutic use of this molecule, such as the following: (i) interaction of cationic delivery systems containing siRNA with plasma proteins leading to formation of aggregates that are either entrapped in the lung endothelial capillary bed or are uptaked by macrophages of the monocyte phagocytic system; (ii) the particle size of delivery system—it was shown that only 50–200 nm particles can cross the permeable endothelium of the neovascularised tumours or inflammation, through the enhanced permeability and retention (EPR) effect; (iii) the delivery system needs to cross the tight network junctions of the extracellular matrix, which is present at the surface of the cells; (iv) overcoming the poor intracellular penetration and sub-cellular localisation of siRNA [116]; and (v) the delivery of siRNA to the CNS must address the problems of neuronal uptake, vesicular escape, and the blood–brain barrier [117]. Research conducted in this area has tried to overcome these barriers, and valuable conclusions have been drawn.

A Janus-type β-CD polycationic amphiphilic derivative was used to deliver siRNA targeting mRNAs into different cancer cell lines, as well as into astrocytes, and to enhance the docetaxel-mediated toxicity in two human prostate cancer cell lines (LNCaP and PC3). The Janus-type amphiphilic derivative was obtained by grafting seven tetraethyleneimine substituents attached to the primary rim of the CD molecule through thioureidocysteaminyl moieties, and fourteen hexanoyl tails at the secondary rim. The amphiphilic CD derivative showed high biocompatibility and protected siRNA from RNAase-mediated degradation, a key property for clinical translation. The CDplexes showed an excellent transfection capability of siRNA targeting mRNAs encoding mitogen-activated protein kinase (p42MAPK) or Ras homologue enriched in the brain (Rheb) in five different cancer cell lines, including two prostate cancer cell lines, as well as in primary astrocytes. In addition, the CDplexes were able to enhance the docetaxel effect in androgen-dependent LNCaP prostate cancer cells, but not in PC3 cell lines, which is a cell line proven to be more resistant to docetaxel. Moreover, the siRNA induced toll-like receptor 3 activation, leading to β-interferon production and caspase activation. Taking all these findings together, the Janus-type amphiphilic CD was proposed as an excellent siRNA carrier for a next generation of molecular vectors incorporating additional functionalities, as well as a general strategy to elicit an immune response against prostate cancer cells [101].

An innovative strategy for treating prostate cancer, based on immunotherapy, was developed by Sun et al. [118] using a cationic amphiphilic β-CD to deliver CSF-1R (colony-stimulating factor-1 receptor) siRNA on a prostate cancer-bearing mouse model. More precisely, NPs were obtained from an amphiphilic cationic beta-CD derivative and siRNA, which were further functionalised with DSPE-PEG-2000, and a macrophage 2 targeting peptide (M2-pep) to maximise specific cellular delivery. Tumour-associated macrophages, which play an important role in the tumour microenvironment, can be reprogrammed from an immunosuppressive M2 phenotype to an immunostimulatory M1 one by downregulation of the CSF-1R signalling pathway, using siRNA.

The resulting NPs were able to reprogramme M2 macrophages by silencing CSF-1 mRNA expression. As a consequence, the reprogrammed M2 macrophages remodelled the tumour microenvironment, reactivating the infiltration of CD4+/CD8+ T cells [118].

O′Mahony et al. synthesised NPs based on a complex between a polycationic amphiphilic β-CD derivative and siRNA to investigate its potential use as a vector for siRNA into neuronal cells. They obtained NPs of less than 200 nm in size, stable in serum, which facilitated high levels of intracellular delivery of siRNA in both immortalised hypothalamic neurons and primary hippocampal neurons, while maintaining at least 80% cell viability. Significant gene knockdown was also achieved, with a reduction in luciferase expression (up to 68%), and of endogenous glyceraldehyde-phosphate dehydrogenase expression (up to 40%) [117].

Godinho et al. loaded siRNA in NPs composed of amphiphilic cationic β-CD derivative with the aim to investigate the potential of these CDs as neuronal carriers, in vitro and in vivo studies. The NPs were stable at 37 °C in artificial cerebrospinal fluid up to 6 h, therefore protecting siRNA from degradation. The CD:siRNA-based NPs were also able to reduce the expression of the huntingtin gene in rat striatal cells and the human Huntington disease primary fibroblasts. The in vivo studies indicated sustained knockdown effects observed in the striatum of the R6/2 mouse Huntington disease model after a single direct NP injection. After repeated brain injections, a selective alleviation of motor deficits in a mouse model was obtained [119].

McCarthy and co-workers synthesised a polycationic β-CD derivative, substituted at C6 with a dodecyl alkyl chain through a thio linker, and at C2 with a aminopropyl-triazole-methyl-oxy, aiming to investigate the efficacy of this CD derivative as a vector for TNF-α siRNA delivery to macrophage cells and to mice with induced acute colitis. siRNA remained intact and stable in simulated colonic fluids, due to the formation of a nanocomplex with polycationic CD derivative. The results also indicated a significant reduction not only in TNF-α levels, but also in IL-6 levels in RAW264.7 (murine macrophage) cells transfected with CD-TNF-α siRNA. In vivo experiments, using mice with induced colitis, showed only a mild amelioration in clinical signs of colitis, but a significant reduction in total colon weight and colonic mRNA expression of TNF-α and IL-6, compared to the control group [114].

#### 4.4.2. Charge Modulation of the Surface of Amphiphilic CD Derivatives to Improve the Delivery of Genetic Material

Given the negative charge of DNA and RNA molecules, cationic carriers are considered most suitable for gene transfer, but exhibit a certain toxicity profile, whereas anionic carriers, which are more biocompatible, do not bind with DNA because of electrostatic repulsion. Therefore, attenuation of the charge density of cationic carriers is desirable to improve the toxicological profile and also to improve the stability of the formulation in salt- and serum-containing media [120].

Furthermore, cationic amphiphilic CDs, due to their cationic nature, are limited for in vivo systemic use, due to the adsorption by serum proteins, followed by clearance by Kupffer cells and other phagocytic cells of the RES [121]. Therefore, research efforts have focused on charge modulation of these CDs-based NPs to improve the gene transfer.

Charge modulation of cationic-based NPs might be achieved by several methods: using PEGylation, using co-formulation of ionic CD and PEGylated CD, and by structural modification of the carrier.

PEGylation of NPs is considered a promising solution for improving the biological characteristics of a delivery system. Polyethylene glycol (PEG) is a polymeric material, neutral and hydrophilic in nature, and PEG functionalisation has been proven to have a stabilising effect, avoiding aggregation. The molecular weight and the grafting density of PEG influence the stability and the clearance rate of NPs. At the cellular level, PEGylation may decrease cellular uptake, influencing the intracellular trafficking and fate of the delivery system and, consequently, of the cargo [121].

Also, at the cellular level, the aggregation of particles may enhance transfection in vitro, as they tend to attach onto cells for association and uptake. When aggregation occurs more slowly, delayed uptake may occur. Although these phenomena were described in vitro, methods to improve formulations to prevent aggregation are required in vivo to enable prolonged circulation and prevent opsonisation [117].

Different strategies for PEGylating CDs-based delivery systems have been employed, such as chemical methods to covalently couple polyethylene glycol chains to the molecule of CD, and also physical methods, such as post-insertion, applied to the inclusion complex.

Guo et al. [122] have synthesised a cationic amphiphilic β-CD derivative (a heptaguanidino-*β*-CD derivative), which was further PEGylated to attach, via the PEG chain, an anisamide group as a targeting ligand, aiming to obtain anisamide-targeted CD nanoparticles, for siRNA delivery to prostate tumours in mice. The formation of the CD:siRNA complex was facilitated by the interaction between the positively charged guanidino group and the negatively charged siRNA. Three types of complexes have been obtained with siRNA, namely with non-PEGylated-CD, with PEGylated-CD, and PEGylated-CD bearing the anisamide group (CD-PEG-AA.siRNA). PEGylation was able to avoid aggregation in the saline solution of the complex, shielding the surface charge of the complex. In addition, the anisamide-targeted complex efficiently protected siRNA against serum nucleases. PEGylated CDs were considerably less toxic than non-PEGylated ones, although the anisamide moiety slightly increased toxicity.

The in vitro studies showed that only anisamide-targeted formulation induced prostate-cell-specific internalisation of siRNA, resulting in a significant knockdown of luciferase. Parenteral administration of the anisamide-targeted formulation in a mouse prostate tumour model demonstrated significant tumour inactivation and reduction in the level of vascular endothelial growth factor (VEGF) mRNA and low toxicity [122].

The research group conducted by O’Mahony used an amphiphilic β-CD derivative bearing a dodecylthio alkyl chain on the primary face to obtain polycationic and neutral PEGylated derivatives, for dual cyclodextrin formulations for the delivery of siRNA. It was shown that cationic CD, when used alone with siRNA, exhibited good transfection properties in cell culture, but the co-formulation with neutral CD derivative lowered the transfection efficiency, partly by masking cationic charge, which is necessary for cell adhesion. Further studies were proposed for proper surface modification of the particles bearing targeted ligands for an effective siRNA delivery [123].

Using the same types of CDs as described in the previous paragraph, Gooding et al. proposed a strategy to reinstate cellular uptake, while simultaneously maintaining the reduced cationic nature of the co-formulation. The strategy was based on using a targeting ligand to potentiate receptor-mediated endocytosis of siRNA NPs, namely the rabies virus glycoprotein (RVG). For this purpose, co-formulation between the cationic amphiphilic β-CD, its PEGylated derivative decorated, or not, with RVG, was proposed for siRNA delivery in human glioblastoma cells. The PEG derivatisation of the CD was preserved, taking into account the disadvantage of obtaining a decreased cellular uptake, due to the masking effect of cationic charge (the cationic charge being necessary for cell adhesion). The RVG peptide was used to increase specific cellular uptake via binding to nAchR (nicotine acetyl cholinesterase) receptor on neuronal cells, as well as for its potential to improve the in vivo biodistribution and decrease the in vivo toxicity profile, related to cationic derivatives. The co-formulation between cationic CD: PEGylated CD: RVG-tagged PEGylated CD and siRNA, at a molar ratio of 1: 1.5:0.5, showed an efficient cellular uptake and a 27% gene-knockdown ability in a model of human glioblastoma cells. Further optimisation was proposed to maximise the gene silence efficiency [121].

In this regard, a delivery system based on cationic amphiphilic CD NPs, loaded with siRNA targeting the huntingtin gene (HTT), and optimised with RVG was reported by Mendonça et al. [124]. The delivery platform was designed to obtain a less invasive delivery system for delivering siRNA into target CNS disease cells. An in vitro co-culture BBB model of Huntington disease (HD) was used.

In order to reduce the cationic nature of the CD, a tertiary amine was incorporated, lowering pKa from 11 to 6.57 (a pKa in the range of 6.2–6.5 was considered optimal for nucleic acid delivery and gene silencing for cationic lipids). More precisely, a β-CD derivative substituted at the primary rim with a dodecylthio group, and at the C2 on the secondary rim, with a dimethyl-amino-butanoyl residue, was complexed with siRNA. Adamantyl-PEG500—RVG (Adm.PEG.RVG) was employed to decorate the NPs. The obtained formulation (with mass ratio of 10:1:5, CD:siRNA:Adm.PEG.RVG) met the attributes for optimum BBB permeability following systemic administration, namely appropriate particle size (169–187 nm), and a slightly positive charge (15–16 mV): no displacement of siRNA was detected with the addition of Adm.PEG.RVG.

All tested formulations, namely CD:siRNA and CD:siRNA:Adm.PEG.RVG, exhibited good biocompatibility and were able to release siRNA into the cytoplasm of the neuronal cells used in the study. The RVG-targeted NPs resulted in greater gene silencing as compared to the untargeted NPs. In fact, the silencing efficiency of the targeted NPs was 20% higher than the untargeted formulation. Two mechanisms were proposed to explain this small difference. Firstly, the variability of the physiological cellular environment may mask the contribution of the RVG peptide (i.e., foetal bovine serum-supplemented culture media); adsorption of proteins in serum might reduce the binding of the targeted formulation to its specific receptors. Secondly, the ligand density and orientation on the surface of the carrier can influence the ligand–receptor engagement. An optimal density is required because an increase in ligand density above the optimal level may lead to a decrease in receptor binding, possibly attributable to a steric hindrance effect [124].

Kont et al. investigated the role of co-formulation between amphiphilic cationic and anionic CDs for siRNA delivery in a model of blood cancer cells. The co-formulation aimed to reduce the cationic charge density and improve the stability and gene knockdown activity. The study also focused on achieving good transfection efficiency, knowing the fact that blood cells have proven to be difficult to transfect compared to other cell types. The cationic CD:siRNA complex was obtained, then co-formulated with an anionic amphiphilic CD derivative. Both formulations, cationic/siRNA and co-formulation, were able to protect siRNA from degradation in the serum, in good agreement with their favourable physicochemical properties. In vitro tests on acute myeloid leukaemia cells (HL-60) indicated a similar level of cellular uptake (60% after 6 h), followed by endosomal escape. The formulations revealed different patterns of endosomal escape (after 6 h for the cationic formulation, and after 24 h for the co-formulation). Tests on HL-60 cells transfected with *KAT2a* siRNA indicated that both formulations significantly reduced mRNA levels related to free *KAT2a* by 29% for the formulation and by 21% for the co-formulation, in the absence of toxicity. The *KAT2a* is an epigenetic modulator, which is overexpressed in all acute myeloid leukaemia types. The following conclusions were drawn: (i) the charge of the particles is not the unique factor influencing the transfection efficiency, because a reduction in overall charge density of the co-formulation did not alter the cellular uptake of the cationic CD:siRNA formulation. In addition, the amphiphilic CD derivative may have a significant role, promoting a lipophilic interaction at cellular level; (ii) the late endosomal escape of co-formulation was expected, since a decrease in cationic charge may lead to a weaker interaction between NPs and endosomal membrane; and (iii) the lower level of gene silencing for co-formulation was attributed to the lower loading capacity [120].

#### 4.4.3. Glyco-Coating of Amphiphilic CDs to Enhance Gene Delivery

Glyco-coating of cationic amphiphilic CDs represents a face-selective functionalisation method to improve the delivery of drugs. Glyco-conjugates can be recognised by specific cell membrane receptors (lectins). The expression of carbohydrate-specific protein receptors depends both on cell type and on cell state [112]. For example, an increased expression of lectin receptors was found in malignant cells, which is very likely involved in cancer metastasis [125].

A series of mannosyl-coated polycationic amphiphilic β-CDs was used to obtain CD: plasmid DNA-based NPs, aiming to evaluate their potential towards vectorised gene delivery. Glyco-coating was used since it usually favours non-specific interactions of cationic surfaces by improving solvation.

The glyco-coated derivatives self-assembled in the presence of plasmid DNA, with formation of nanometric CDplexes that exhibited transfection capabilities in RAW 264.7—mouse leukaemic monocyte macrophage cells, known to express moderate mannose receptors. Further studies indicated that the bio recognisable mannosyl moieties were exposed at the surface in the CDplexes, and provided the system with molecular recognition abilities towards mannose-specific lectins, including concanavalin A and the human macrophage mannose receptor. Macrophage adhesion studies demonstrated the involvement of unspecific binding, attributed to electrostatic interactions with negatively charged cell membrane components. The study pointed out that a proper balance between the shielding role of the sugar moiety at the surface of the particle and the biorecognisable one is critical for cell-specific targeting [112].

Finally, a summative situation of the above-discussed studies regarding the use of amphiphilic CDs delivery system, with focus on their key functionalities and experimental conclusions, is presented in Table 3.

## 5. Conclusions and Future Perspectives

### 5.1. Conclusions

Cyclodextrins, since their discovery in the late 19th century, have gained an important role in pharmaceutics and medicine, for enhancing the efficacy of drugs, and for their own therapeutic potential.

The present review, centred on literature reports, described the constant ascent into pharmaceutical and medicinal applications of amphiphilic cyclodextrins.

Amphiphilic CDs gained their usefulness in overcoming the limits of natural CDs in pharmaceutical applications, due to an enhanced interaction with drug molecules and bio-membranes, improved release profiles of entrapped drugs and cytotoxicity, and, not least, their self-assembling properties with formation of colloidal supramolecular aggregates.

On the one hand, there is an ascendant trend in repurposing old drugs aiming to increase their efficacy, including redesigning through cyclodextrins, which is less expensive than the discovery of a new drug.

On the other hand, recognising the pivotal role of amphiphilic CDs as promising tools in nanomedicine, the pursuit of innovative derivatives and techniques to selectively modulate their pharmaceutical applications has gathered considerable attention in research.

### 5.2. Future Perspectives

Numerous ongoing research studies are exploring the potential of amphiphilic CDs in various therapies, investigating new applications, modifications of their structure, and improved delivery systems. Future advancements are expected, as amphiphilic CDs offer multiple possibilities for modification and supramolecular system construction to meet drug therapeutic needs, also allowing for more precise and individualised treatments.

Amphiphilic CDs show great promise in cancer therapy by promoting tumour targeting, an enhanced drug-mediated toxicity, and increased stability of the drug in various physiological media. Due to their specific architecture, which allows for a multi-domain mediated interaction with the drug, these particular CD derivatives are also suitable for dual anticancer therapy.

Amphiphilic CDs are being explored in gene therapy as an alternative to viral vectors. Their role in non-viral delivery systems is valuable, being endorsed by their favourable toxicological profile and their ability to improve stability and cellular uptake of genetic material. Moreover, their efficacy was demonstrated on CNS diseases, which could revolutionise treatments in this area.

Although the scale-laboratory methods of preparing amphiphilic CD-based delivery systems are successfully employed, more studies, looking to transfer these efficient laboratory methods to the factory-scale production, are necessary.

Studies to clear up the regulatory issues and safety concerns are also required for advancing clinical translation. So far, research in the field has shown a good toxicological profile of amphiphilic CDs, thus enhancing the advancements for obtaining commercially available products based on amphiphilic cyclodextrins.

## Figures and Tables

**Table 1:** Structural and physicochemical properties of natural CDs [19,20].

Characteristics	α-CD	β-CD	γ-CD
Number of glucose units	6	7	8
Molecular weight	972	1134	1296
Number of OH groups/molecule	18	21	24
Cavity diametre (Å)	4.7–5.3	6–6.5	7.5–8.3
Torus height (Å)	7.9 ± 0.1	7.9 ± 0.1	7.9 ± 0.1
External diametre (Å)	14.6 ± 0.4	15.4 ± 0.4	17.5 ± 0.4
Water solubility (g/100 mL) at room temperature	14.5	1.85	23.2
[α]_D_ 25 °C	150 ± 0.5	162 ± 0.5	177.4 ± 0.5
Melting temperature range (°C)	255–260	255–265	240–245
Water molecules in the cavity	6	11	17

**Table 2:** Toxicological data of some amphiphilic CDs.

Amphiphilic CD Derivative	Toxicological Data	References
β-CDC6 and 6-*O*-Capro-*β*-CD (amphiphilic β-CD derivatives modified on the secondary face, and, respectively, on the primary face with 6C aliphatic chains)	Non-toxic on mouse fibroblast L929 cells at 1:128 dilution	[68]
Folate-conjugated amphiphilic β-CD derivatives	Cell viability on L929 mouse fibroblast cell line in range of 80–100% at any concentration, through 24 or 48 h incubation	[54]
Amphiphilic β-CD derivative random substituted with C12 alkyl chains	Well-tolerated in acute toxicity studies in mice, by i.v. administration, at a dose > 2000 mg/kg	[53]
6-*O*-Capro-*β*-CD (β-CD derivative modified on the primary face with 6C aliphatic ester) nanospheres/ nanocapsules	Concentration-dependent haemolysis (on human blood samples); Haemolysis of 40–60% (for nanospheres, at maximum concentration of 50 mM, used in the experiment) and 60–80% (in case of nanocapsules)	[45]
	% of cell viability on L929 mouse fibroblast cell line, upon dilution, was in the range of 40–70%	

**Table 3:** Summative table of the discussed studies regarding the use of amphiphilic cyclodextrin nanosystems.

	Compound	Drug Delivery System	Key Functionalities	Experimental Conclusions	Ref.
1	Tamoxifen citrate	Nanospheres and nanocapsules of amphiphilic β-CD modified on the secondary face with 6C aliphatic esters	Pre-loaded NPs were prepared directly using pre-formed tamoxifen citrate: β-CDC6 complex (1:1) with no further drug being added to the system during preparation; High-loaded NPs were prepared directly using pre-formed tamoxifen citrate: β-CDC6 complex (1:1) with further drug being added to the system during preparation; Conventional-loaded NPs were prepared by addition of drug solution to organic phase during preparation; For the preparation of nanocapsules, the organic phase was Miglyol 812^®^ (a neutral oil composed of triglycerides of the fractionated vegetable fatty acids C8 and C10) dissolved in acetone, and for the preparation of nanospheres, the organic phase was acetone.	Particle size and zeta potential values indicated that tamoxifen citrate was adsorbed to the nanospheres’ surface and entangled in aliphatic chains aligning the surface of the NP, while in the case of nanocapsules, the drug was mainly encapsulated within the oily core, with only a small amount on the surface aligned with free –OH groups of the β-CDC6 molecule; The importance of using pre-formed inclusion complexes: (a) increased entrapment efficiency—about 2-fold in case of nanospheres, and 3-fold in case of nanocapsules, as compared to the conventional-loaded NPs, (b) the delay of drug release—(i) a complete release of drug within 15 min for conventionally loaded nanospheres, while for pre-loaded nanospheres and high-loaded ones, the complete release was achieved within 6 h and 2 h, respectively, (ii) the same phenomenon was observed for nanocapsules, with the observation that both pre-loaded and high-loaded systems showed a release profile extended to a period of 6 h; No toxicity was observed for unloaded NPs against MCF-7 breast cancer cells, while loaded NPs exerted an equally efficient cytotoxic activity as tamoxifen citrate (solution in acetone).	[44]
2	Paclitaxel	Nanospheres and nanocapsules of amphiphilic 6-*O*-Capro-*β*-CD	High-loaded NPs were prepared directly using pre-formed paclitaxel: 6-*O*-Capro-*β*-CD complex (1:1 molar ratio) with further drug solution being added to the system during preparation; Conventional-loaded NPs were prepared by addition of drug solution (200 μL) in organic phase during preparation; To prepare nanocapsules of amphiphilic CD, Miglyol 812^®^ (50 μL) in organic phase (acetone) was added; The highest possible concentration of NPs and mixture of commercial vehicle cremophor/ethanol were used in order to represent the most exaggerated conditions.	Stable nanospheres and nanocapsules of 150 nm diametre and 500 nm diametre, respectively, with up to 65% entrapment efficiency for paclitaxel were obtained; The NPs were characterised by controlled release profiles of biphasic nature with complete in vitro release in 24 h; The blank NPs showed significantly less haemolytic effect than the blank cremophor/ethanol mixture; nanocapsules were slightly more haemolytic than nanospheres, attributed to the presence, in the case of nanocapsules, of the Miglyol 812 as oil phase; Blank NPs showed a favourable toxicological profile against L929 mouse fibroblast cell line, as compared to cremophor/ethanol mixture, underlying the usefulness of CD-based carrier in avoiding the self-toxicity of the vehicle (carrier); The amphiphilic CD-based nanoparticle system was able to maintain the physical stability of paclitaxel, avoiding the recrystallisation process and precipitation in aqueous medium, a phenomenon that is possible at parenteral administration, resulting in severe necrosis at the injection site and impairment of clinical efficacy of the drug.	[45]
3	-	Polycationic amphiphilic β-cyclodextrin (β-CDC6) nanoparticles	A belt of 7 primary amino groups on the CD primary face (protonated at physiological pH) for increased cellular binding and uptake by negative cell membrane; A cluster of 14 hexanoyl chains on the CD secondary face.	Spherical-shaped NPs, 75 nm in diametre, +61 mV surface charge (aqueous medium), conferring suitable characteristics for hepatic targeting and improved cellular interaction; Dose-dependent and selective cytotoxicity on HepG2 cell culture and no cytotoxic effect on L929 fibroblast cells; Activation of apoptosis correlated with Caspase-3/7 activation, inhibition of Survivin (an inhibitor of apoptosis in cancer cells) in HepG2 cells; Depletion of cholesterol in HepG2 cells (which was correlated with the suppression of Survivin expression), changes in cell morphology and viability; Overcoming drug resistance in HepG2 cells due to alleviation of P-gp transporter protein.	[91]
4	Camptothecin	Nanospheres of amphiphilic 6-*O*-Capro-*β*-CD Nanospheres of amphiphilic β-CDC6	6-*O*-Capro-*β*-CD amphiphilic derivative modified on the primary face with substitution of C6 linear alkyl chains and hydrophobic ester bond; β-CDC6 amphiphilic derivative modified on the secondary face with 6C aliphatic esters; high-loaded nanospheres were prepared using pre-formed inclusion complexes of camptothecin and amphiphilic CD derivative, and by further dissolving an additional amount of drug in organic phase.	Amphiphilic 6-*O*-Capro-*β*-CD had a higher loading capacity than amphiphilic β-CDC6 derivative, suggesting that leaving the secondary face unsubstituted reduces the steric hindrance and enables drug loading efficiency; Releasing of the drug in its stable and biologically active lactone form from both types of amphiphilic CD NPs for more than 12 days for 6-*O*-Capro-*β*-CD NPs and 6 days for β-CDC6 NPs, with a more vigorous burst effect in the case of β-CDC6 NPs, resulting in 50% cumulative release of drug after 5 h in contrast to 24 h in the case of 6-*O*-Capro-*β*-CD NPs; Anticancer efficiency of amphiphilic CD NPs determined on MCF-7 human breast adenocarcinoma cells revealed that camptothecin was more effective when encapsulated as compared to camptothecin solution in DMSO, with better results observed in the case of 6-*O*-Capro-*β*-CD; Blank amphiphilic CD NPs showed a favourable toxicological profile against L929 mouse fibroblast cells.	[68]
5	Paclitaxel	Paclitaxel-loaded folate-targeted nanoparticles based on amphiphilic cyclodextrin derivatives for intravenous route	Folate-conjugated amphiphilic CD derivatives modified on the secondary side/primary side (FCD-1/FCD-2) with substitution of C6 linear alkyl chains by esterification, and carrying the folate residue on the substituted face at the end of C6 linker chain; The folate residue enables active targeting folate-positive breast tumour cells; Paclitaxel-loaded nanospheres were prepared using nanoprecipitation technique in which folate-CD and paclitaxel solution were added in the organic phase; Blank nanospheres were also prepared using nanoprecipitation technique.	Blank and paclitaxel-loaded folate-conjugated amphiphilic NPs were characterised by regular spherical shape with a narrow and unimodal size distribution; Encapsulation efficiency was 60.4% for FCD-1 NPs (120.8 μg drug/mg CD) and 35.2% FCD-2 NPs (70.4 μg drug/mg CD); In vitro release studies in pH 7.4 phosphate-buffer saline solution with 0.1% Tween 80 as medium, revealed for FCD-1 NPs, an initial burst effect, with 44% of amount of paclitaxel being released during the first hour, followed by a liberation of 90% of total amount of paclitaxel, in a steady manner, in the next 24 while for FCD-2 NPs, an initial burst release of 72% of paclitaxel was observed during the first 1 h, followed by a steady release for the next 6 h (95% of loaded paclitaxel was released at the end of the experiment); FCD-1 and FCD-2 blank NPs exerted no toxicity on L929 mouse fibroblast cell line, through 24 or 48 h (cell viability range 80–100%); FCD-1 and FCD-2 blank NPs applied onto T-47D and ZR-75-1 human breast cancer cells (FR-α being expressed by these types of cells) exhibited a more efficient cellular uptake in the case of FCD-1 than that of FCD-2, in both cell lines; On the T-47D cell line, both paclitaxel-loaded FCD-1 and FDC-2 NPs exerted comparative anticancer efficacy as compared to that of paclitaxel solution alone, while in the case of ZR-75-1 cells, both types of paclitaxel-loaded NPs showed significantly increased anticancer effect; the effect was considerably clearer in the case of low concentrations of FCD-1 NPs.	[54]
6	Tamoxifen citrate	Nanovesicles based on amphiphilic β-CDC12	Substitution of primary and secondary rims of β-CD with C12 hydrophobic alkyl chains, from lauric acid, via ester bonds; Amphiphilic cyclodextrin contains approximately 7 alkyl chains on both the primary and the secondary rim of β-CD (lauric acid: β-CD molar ratio was 15:1); Surfactant was used in order to optimise the particle size and stability of nanovesicles.	Synthesised amphiphilic CDs had the capability of self-assembling to form spherical nanovesicles in aqueous medium; The surfactant Kolliphor P407 (0.5% *w*/*v*) (Poloxamer P407, Pluronic F-127) optimised the particle size and the stability of nanovesicles, as compared to those prepared without surfactant; A stable complex was obtained between amphiphilic β-CD and tamoxifen citrate, helping to retain tamoxifen in nanovesicles and overcoming the disadvantage of precipitation of the drug (which is a common problem for liposomal formulations); The encapsulation efficiency of tamoxifen citrate was 94%; Amphiphilic CDs helped in the reduction in burst release of tamoxifen citrate and maintained the prolonged release, approximately 75% of the drug being released over a period of 48 h in both dissolution media (pH 6.5—intratumoural pH, and pH 7.4—blood plasma pH); In vitro haemolysis study showed that amphiphilic CDs revealed no haemolytic effect, as compared to the parent β-CD, probably due to the self-assembling property, and no genotoxicity; In vitro cell cytotoxicity studies on MCF-7 cells showed that blank amphiphilic CD nanovesicles were highly biocompatible, and tamoxifen citrate-loaded nanovesicles showed a significantly enhanced growth inhibitory effect than the freely diffusible tamoxifen solution at lower concentrations; In vitro cell uptake studies revealed a caveolae-dependent cell internalisation pathway of amphiphilic β-CD nanovesicles in MCF-7 cells; Acute toxicity studies in mice demonstrated that amphiphilic β-CD was well-tolerated by intravenous route, at doses as high as 2000 mg/kg; The pharmacokinetic profile of tamoxifen citrate encapsulated in nanovesicles was improved, and a 3-fold higher AUC was observed; The pharmacokinetic properties suggested that the amphiphilic β-CD nanovesicles assured a longer retention of the drug in plasma, and, therefore, for entering tumor tissues through enhanced permeation-retention effect.	[53]
7	Docetaxel and Zinc (II)–phthalocyanine	Nanovesicles based on heptakis (2-oligo(ethylenoxyde)-6-hexadecylthio-)-*β*-CD (SC16OH)	NPs containing docetaxel and Zinc (II)–phthalocyanine with theoretical loading of 5% and 0.2%, respectively, were prepared by the emulsion-solvent evaporation technique; all three components were co-dissolved in organic medium (dichloromethane/tetrahydrofuran 9:1 *v*/*v*), and the mixture was added to water and then sonicated. The organic solvent was eliminated by mechanical stirring, NPs were re-dispersed in water, freeze-dried, and kept at 4 °C.	The NPs showed a modal size distribution, a hydrodynamic diametre of approx. 200 nm, and a negative zeta potential, and a satisfactory entrapment efficiency of both drugs at a specific mass ratio; The docetaxel was entrapped in the hydrophobic portion of CD, preferring the space occupied by interdigitated hydrophobic chains of two adjacent CDs, while Zinc (II)–phthalocyanine interaction with CDs involved both oligo-ethylenglycol moieties and thioalkyl moieties; Zinc (II)–phthalocyanine was entrapped as a monomer (which is a fundamental prerequisite to show photodynamic effect) in the carrier, thus maintaining a fairly high propensity to photogenerate oxygen singlet; The dissolution profile of NPs allowed a sustained and modulated release of entrapped drugs; A very low haemolysis was observed at the highest investigated concentrations of NPs; In HeLa cells, photodamage due to Zinc(II)–phthalocyanine enhanced the cytotoxic effects of docetaxel.	[70]
8	Short interfering RNA (siRNA)	Folate-targeted cationic amphiphilic cyclodextrin-based NPs	Cationic amphiphilic cyclodextrin complexed with siRNA; The amphiphilic SC_12_CDclickpropylamine cyclodextrin (cationic groups on the secondary face and C_12_ on the primary face) with great therapeutic potential for siRNA delivery; DSPE-PEG_5000_–folate and GALA peptide were post-inserted into pre-formed CD-siRNA complexes; Incorporation of PEG of various lengths may increase the stability of cyclodextrin-based NPs and may increase the t_1/2_of siRNA in vivo; DSPE-PEG_5000_ post-insertion into pre-formed amphiphilic CD-siRNA NPs may result in a more spherical structure; GALA may improve endosomal escape of cationic NPs, thus improving siRNA delivery into cells; DSPE-PEG_5000_-folate incorporated into pre-formed CD-siRNA complex for improving pharmacokinetic profile of siRNA.	NPs showed favourable physicochemical properties (spherical, with approx. 200 nm in size), and capable of resisting aggregation for up to 24 h in salt-containing medium; NPs efficiently bound siRNA; NPs protected siRNA from serum nucleases due to the presence of cyclodextrin plus PEG; NPs displayed folate-mediated uptake in prostate cancer cell lines PC3 and LNCaP cells, highlighting the benefit of targeting NPs with folate for the treatment of prostate cancer; NPs showed a low cytotoxic profile in LNCaP and PC3 cells compared with untreated controls; NPs complexed with luciferase siRNA showed a specific knockdown of the luciferase reporter gene in vitro due to addition of GALA to the formulation; NPs highlighted a superior silencing ability to targeted genes (NRP1 and ZEB1) in metastatic prostate cancer PC3 cells as compared to control, and reduced infiltration; (ZEB1 is a marker of epithelial to mesenchymal transition, a process responsible for metastasis of different carcinoma types; NRP1 is a therapeutic target responsible for migration of cancer cells).	[103]
9	Short interfering RNA (siRNA)	PEGylated cationic amphiphilic cyclodextrin-based NP containing siRNA, tagged with a central nervous system-targeting peptide derived from the rabies virus glycoprotein (RVG), at a molar ratio of 1:1.5:0.5 (cationic cyclodextrin/PEGylated cyclodextrin/RVG-tagged PEGylated cyclodextrin)	A low molecular weight PEG with high density was incorporated in co-formulation to reduce the cationic charge; The rabies virus glycoprotein (RVG) was chosen as a ligand to reinstate cellular uptake while maintaining the reduced cationic nature of the co-formulation; RVG specifically binds to nicotinic acetylcholine receptors on neuronal cells, thus increasing cellular uptake and providing the ability to cross the blood–brain barrier.	Incorporation of PEG in CD-based siRNA nanocomplex reduced surface charge, stabilised the NPs, and lowered the zeta potential. The nanocomplex protected siRNA against nucleases degradation for more than 4 h. An optimal amount of RVG-tagged PEGylated cyclodextrin is required to achieve a maximum cellular uptake. The surface density and smaller molecular weight of PEG_500_ and the residual cationic charge were favourable for achieving the maximum cellular uptake in U87 human glioblastoma cells. CD-based siRNA nanocomplex showed a favourable toxicological profile in U87 human glioblastoma cells. The RVG-specific acetylcholine receptors predominantly facilitated the cellular uptake in U87 human glioblastoma cells, and highlighted the receptor-mediated endocytosis process. An enhanced gene-silencing effect was achieved by CD-based siRNA nanocomplex, compared to the corresponding untargeted PEGylated formulations.	[121]
10	Short interfering RNA (siRNA)	Co-formulation of cationic CD:siRNA:anionic CD with a mass ratio of 8.5:1:5.3 The anionic amphiphile is a β-CD derivative, with lipid chains (SC12) grafted on the secondary side and a negative moiety on the primary side The cationic amphiphile is a β-CD derivative with lipid chains (SC12) on the primary side and a primary amine function on the secondary side	NPs were obtained using pre-formed cationic CD:siRNA complex and anionic CD.	The co-formulation exhibited a surface charge of 24 ± 6 mV, size of 161 ± 14 nm, and PDI 0.16 ± 0.04, and 75% encapsulation efficiency, with promising in vitro uptake, capacity to escape the reticulo-endothelial system (RES), and uniform particles; The addition of amphiphilic anionic CD to the formulation positively impacted the surface charge of the NP, by reducing the pKa to 7 ± 0.35 (it has been reported that materials with pKa values around 6.5 are optimal for non-viral delivery of nucleic acids); There was no displacement of siRNA on addition of the anionic CD at the mass ratio studied; The examination of size and morphology indicated a structural arrangement with the integration of both lipidic functionalities, forming a bilayer-like structure; The co-formulation was able to protect siRNA from enzymatic degradation in the serum; The siRNA internalisation in HL-60 cells (isolated from the peripheral blood of an acute myeloid leukaemia patient, with overexpression of the epigenetic modulator KAT2a) was confirmed with confocal microscopy; Endo-lysosomal escape appeared at the time of 24 h and may be attributed to a lower electrostatic interaction with endogenous anionic components of the endosomal membrane, due to pKa value of co-formulation, of approx. 7; CD-NP co-formulation significantly reduced mRNA levels relative to free *KAT2a* by 21 ± 2%, in the absence of toxicity in HL-60 cells (previously transfected with *KAT2a* siRNA.	[120]
11	Short interfering RNA (siRNA) targeting mRNA encoding mitogen-activated protein kinase (p42-MAPK) or Ras homologue enriched in brain (Rheb)	CDplexes of polycationic amphiphilic β-CD derivative	Multi-head/multi-tail Janus-type β-CD derivative that combines seven tetraethyleneimine branches attached to the primary rim, through thioureidocysteaminyl connectors, and fourteen hexanoyl tails at the secondary face.	CDplexes were formed having a round shape, an average hydrodynamic diametre of 100 nm, and positive ζ-potential values of 25.4 mV. Hydrophobic interactions involving the lipid domains favoured a lamellar internal order, consisting of successive layers of β-CD derivative bound together by siRNA molecules. The CDplexes protected siRNA from degradation by RNAses. No toxicity was found in the case of β-CD derivative up to 5 μM when used on different cell types, namely rat (C6), mouse (GL261), and human (U87) glioblastoma cell lines, primary rat astrocytes. The β-CD derivative was able to transport siRNA to the interior of the cells, and the complex was able to escape from endosomes into the cytoplasm. The CDplexes caused a significant decrease in both p42-MAPK and Rheb protein levels in C6 rat, GL261 mouse, and in human U87 glioblastoma cell lines, and in p42-MAPK or Rheb protein levels in human prostate cancer LnCaP or PC3; Moreover, a 65% decrease in p42-MAPK protein levels and a 75% decrease in Rheb protein levels were observed in rat astrocytes, a primary culture usually exceedingly difficult to transfect. In human LnCaP cells, a model for the early stages of androgen-dependent prostate cancer, the CDplexes were able to knock down the p42-MAPK and Rheb proteins and to enhance the efficacy of docetaxel. This was not the case for human PC3 cells, a model for more advanced, castration-resistant prostate cancer, attributed to the fact that this type of cell lines are more resistant to taxanes compared to LNCaP cells. The siRNA induced toll-like receptor 3 activation, leading to β-interferon production and caspase activation, in LNCaP cells.	[101]

## Data Availability

No new data were reported. Further inquiries should be addressed to the corresponding authors.

## Abbreviations

The following abbreviations are used in this manuscript:

BBB	blood–brain barrier
CD	cyclodextrin
CNS	central nervous system
CRISPR	clustered regularly interspaced short palindromic repeats
DNA	deoxyribonucleic acid
pDNA	plasmid deoxyribonucleic acid
DSPE-PEG	1,2-Distearoyl-*sn*-glycero-3-phosphoethanol-amine-poly (ethylene glycol)
HeLa	cervical cancer cells
HepG2	hepatocellular carcinoma cell line
HP-*β*-CD	hydroxypropyl-beta-cyclodextrin
HD	Huntington disease
HTT	huntingtin gene
EPR	enhanced permeability and retention effect
FA	folic acid
FR	folic acid receptor
IC	inclusion complex
IC_50_	half-maximal inhibitory concentration
IL	interleukine
L929	mouse fibroblast cell line
MAPK	mitogen-activated protein kinase
MCF-7	Michigan Cancer Foundation-7 human breast adenocarcinoma cell line
NP	nanoparticle
MB49	murine bladder carcinoma cell line
PC	prostate cancer
PCL	poly-ε-caprolactone
PDI	polydispersity index
PEG	polyethylene glycol
PLGA	poly(lactide-co-glycolide)
RES	reticuloendothelial system
Rheb	Ras homologue enriched in brain
RNA	ribonucleic acid
RNAi	ribonucleic acid interference
mRNA	messenger ribonucleic acid
siRNA	small interfering ribonucleic acid
RVG	rabies virus glycoprotein
TNF	tumour necrosis factor
